# Heart-brain axis pathophysiological understanding and clinical impact

**DOI:** 10.3389/fcvm.2026.1681715

**Published:** 2026-04-22

**Authors:** Federico Vancheri, Sergio Vancheri, Giovanni Longo, Edoardo Vancheri, Michael Y. Henein

**Affiliations:** 1 Department of Internal Medicine, S. Elia Hospital, Caltanissetta, Italy; 2 Interventional Neuroradiology Department, Besançon University Hospital, Besançon, France; 3 Cardiovascular and Interventional Department, S. Elia Hospital, Caltanissetta, Italy; 4 Department of Neurology, Hôpital Lariboisière, Paris, France; 5 National Heart and Lung Institute, Imperial College, London, United Kingdom

**Keywords:** heart-brain axis, myocardial infarction, stroke, stroke-heart syndrome, takotsubo (stress) cardiomyopathy

## Abstract

The heart and brain are anatomically and functionally interconnected through nervous and humoral feedback mechanisms. Under physiological conditions, the heart-brain axis helps maintain cardiovascular and cerebral homeostasis. Pathology affecting one organ can profoundly impact the other, significantly worsening prognosis. The term *stroke–heart syndrome* refers to cardiovascular complications following acute ischemic stroke, including myocardial injury, infarction, ventricular dysfunction, arrhythmias (e.g., atrial fibrillation), heart failure, takotsubo syndrome, and sudden cardiac death. Brain damage-induced cardiac injury arises from a complex interplay of neuroinflammation, systemic immune activation, sympathetic-immune interactions, catecholamine toxicity, endothelial dysfunction, and gut–brain–heart axis involvement. Conversely, cardiac conditions, including myocardial ischemia, heart failure, and atrial fibrillation, are associated with an increased risk of stroke and cognitive decline. Myocardial ischemia can initiate systemic inflammation and neuroinflammation through sympathetic overdrive and platelet activation. Heart failure causes cerebral hypoperfusion and high thromboembolic risk, and atrial fibrillation promotes thrombus formation due to blood stasis. Atrial dysfunction and prothrombotic states may also occur independently of arrhythmia. This review summarises current evidence on the pathological interactions within the heart–brain axis, in the context of stroke, mental stress, ischemic heart disease, heart failure, and atrial fibrillation.

## Graphical Abstract

**Figure:**
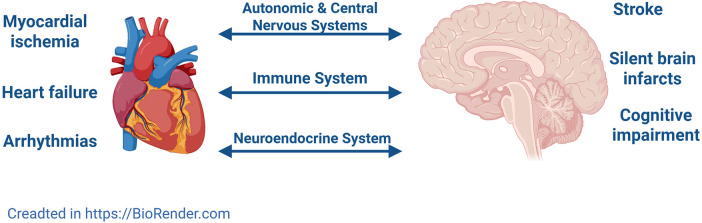

## 1 Introduction

The diagnosis and treatment of cardiovascular disease (CVD) have traditionally focused on the primary clinical syndrome, such as stroke, myocardial infarction (MI), heart failure (HF), or arrhythmias, treating them as isolated disorders affecting either the heart or the brain. Although cardiac and cerebrovascular diseases share many underlying risk factors, including hypertension, diabetes, smoking, and dyslipidaemia, recent evidence suggests that pathological conditions involving both the heart and brain are not merely coincidental occurrences of separate diseases. Rather, they frequently arise from reciprocal and dynamic interactions between the cardiovascular (CV) and the nervous systems (1–4).

The heart and the brain are anatomically and functionally interconnected through complex neural and humoral pathways characterized by bidirectional feedback mechanisms mediated by the autonomic nervous system, hormones, and the immune responses. Under physiological conditions, this integrated network enables both systems to adapt to internal demands and external stimuli. However, pathological alterations in one organ can significantly impair the function of the other, often leading to adverse clinical outcomes.

Both ischemic and hemorrhagic stroke, as well as psychiatric disorders, such as depression and dementia, can disrupt cardiac neural regulation, resulting in myocardial ischaemia, left ventricular (LV) dysfunction and arrhythmias (5–10). Conversely, acute and chronic coronary artery disease (CAD), HF, atrial fibrillation (AF) and invasive cardiac interventions are associated with an increased risk of stroke and vascular dementia (11–15)**.**

Despite this growing body of evidence, current risk stratification models do not incorporate neurocardiac parameters. Contemporary cardiovascular and stroke guidelines rely primarily on traditional clinical risk factors and organ-specific biomarkers. Consequently, no validated framework currently exists for integrating heart–brain interactions into routine prognostic assessment.

In this review, we synthesise contemporary evidence on the anatomical substrates, molecular mechanisms, and clinical manifestations of the heart–brain axis across major pathological conditions, including stroke, psychological stress, myocardial ischaemia, HF, AF, and invasive cardiac procedures. The novelty of this review lies in its comprehensive, bidirectional perspective, emphasizing shared inflammatory, autonomic, and microvascular mechanisms that unify cardiovascular and neurological diseases within a single pathophysiological paradigm.

By integrating anatomical, molecular, and clinical evidence, this review aims to support the development of more comprehensive prognostic frameworks. Shared pathophysiological pathways provide a basis for novel biomarker panels and risk models that go beyond organ-specific assessments. Incorporating neurocardiac parameters such as heart rate variability, troponin dynamics, and combined cardiac and cerebral imaging markers into composite scores may improve early identification of patients at risk for heart–brain complications. This bidirectional paradigm also has implications for trial design and guideline development. Cardiovascular and neurological trials are largely conducted in parallel, with few shared endpoints. Future studies should include both cardiac and neurological outcomes, such as cognitive function, silent brain infarctions, and arrhythmia burden, to better reflect heart–brain interactions. Guideline should likewise consider integrated management pathways for high-risk groups, such as patients with stroke-heart syndrome, heart failure with cognitive impairment, or atrial fibrillation with silent brain ischaemia, as a practical step toward bridging cardiology and neurology in clinical care.

## 2 Functional anatomy of the heart-brain axis

### 2.1 Brain-to-heart pathways

**Figure 1:**
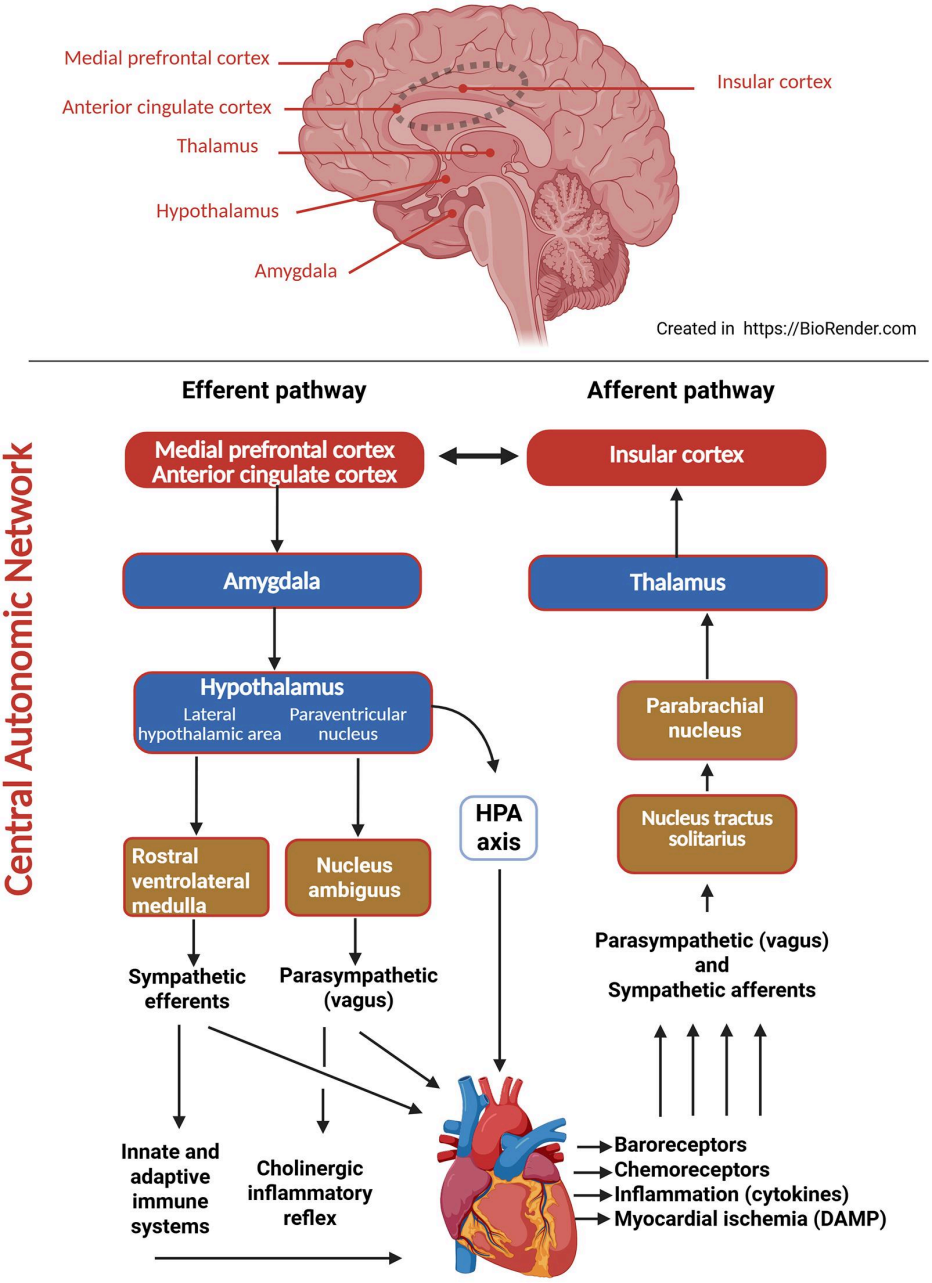
Bidirectional neural, immune, and neuroendocrine network of the heart-brain axis. The upper panel illustrates the principal cortical (red box) subcortical (blue box), and brainstem (brown box) structures forming the CAN involved in cardiovascular regulation. The lower panel depicts the bidirectional efferent and afferent pathways linking the brain and heart. Along the **efferent pathway** (left), cortical regions, including the medial prefrontal cortex and anterior cingulate cortex, process emotional and cognitive input and project via the amygdala to the hypothalamus. The paraventricular nucleus of the hypothalamus activates the HPAS axis and coordinates descending autonomic output. Sympathetic efferents originate from the RVLM, while parasympathetic efferents arise from the nucleus ambiguus via the vagus nerve. Both limbs of the ANS converge on the heart, modulating cardiac electrophysiology, myocardial contractility, and coronary tone. Sympathetic signalling also activates the innate and adaptive immune systems through lymphoid organ innervation, linking neurogenic stress to systemic inflammation. Parasympathetic activity exerts an anti-inflammatory effect, known as cholinergic inflammatory reflex. Along the **afferent pathway** (right), baroreceptors, chemoreceptors, inflammatory mediators (cytokines), and DAMP released during myocardial ischaemia transmit sensory signals from the heart to the NTS. These signals are relayed sequentially through the parabrachial nucleus and thalamus to the insular cortex, enabling integrated haemodynamic regulation and closing the brain-heart feedback loop. CAN, central autonomic network; HPA, hypothalamic-pituitary-adrenal axis; RVLM, rostral ventrolateral medulla; ANS, autonomic nervous system; DAMP, damage-associated molecular patterns; NTS: nucleus tractus solitarius.

The brain and the heart are interconnected through the central nervous system (CNS), the autonomic nervous system (ANS), and immune system. Efferent pathways from the brain to the heart originate within the central autonomic network (CAN), an anatomically and functionally defined network comprising cortical, subcortical, brainstem, and spinal regions that integrates central and peripheral nervous system input (16–19). The CAN coordinates autonomic output by processing afferent sensory input and generating efferent responses that regulate CV function, transmitting signals to the heart and vasculature via the ANS while receiving sensory feedback through afferent pathways. The sympathetic and parasympathetic branches of the ANS exert direct effects on cardiac electrophysiology, myocardial contractility, vascular tone, and immune response [Figure 1, (20)]. Dysregulation within these pathways can contribute to cardiac dysfunction and adverse clinical outcomes.

#### 2.1.1 Anatomy and clinical relevance of the CAN

##### 2.1.1.1 Cortical structures

The cortical component of the CAN play a fundamental role in modulating autonomic activity during emotional and cognitive processes (2, 18, 21). The principal cortical regions involved include the insular cortex, the medial prefrontal cortex, the orbitofrontal cortex, the anterior cingulate cortex, and the posterior cingulate cortex.

The insular cortex (insula) is a key CAN component functionally linked to the limbic system, located within the lateral sulcus beneath the frontal, parietal, and temporal lobes. Ischemic lesions involving this region have been associated with an increased risk of cardiac complications (9, 22). Due to its vascular supply from the middle cerebral artery, the insula is frequently affected in ischemic stroke. Functional lateralisation has been observed in autonomic control, with the right insula predominantly associated with sympathetic activity and the left insula more closely linked to parasympathetic regulation (23).

Beyond autonomic regulation, the insula integrates autonomic, neuroendocrine, and inflammatory viscerosensory feedback into subjective emotional states (interoception) (24). Acute emotional stress may alter insular modulation of sympathetic and parasympathetic tone, leading to increased plasma catecholamine concentrations and a heightened risk of arrhythmias, including AF, complex ventricular and supraventricular arrhythmias, sudden cardiac death, and direct myocardial injury, such as myocytolysis and stress-induced (Takotsubo) cardiomyopathy (25, 26).

The medial prefrontal cortex, located in the medial frontal lobe, and orbitofrontal cortex, situated on the ventral surface of the frontal lobes, are involved in the cognitive appraisal and regulation of emotional responses. These regions exert inhibitory control over excessive sympathetic activation and facilitate parasympathetic recovery following stress.

The anterior cingulate cortex (ACC) located on the medial surface of the frontal lobes superior to the corpus callosum, integrates emotional, cognitive, and interoceptive information to modulate autonomic outflow. Through connections with limbic structures, the hypothalamus, and brainstem nuclei, it regulates sympatho-vagal balance during stress. Dysfunction in this region may promote autonomic imbalance and increase susceptibility to stress-related CV disease.

The posterior cingulate cortex located in the medial parietal lobe, integrates interoceptive, sensory, and cognitive inputs and contributes to autonomic modulation.

##### 2.1.1.2 Subcortical structures

Subcortical structures, including the amygdala, hypothalamus, and thalamus, integrate emotional and sensory information with autonomic and neuroendocrine responses to maintain cardiovascular homeostasis (27).

The Amygdala, a paired limbic structure in the medial temporal lobe, mediates the relationship between psychological stress and CV disease by processing emotional and environmental stimuli (28). Through its connections with the insula and hypothalamus, the amygdala modulates CAN activity and influences autonomic control of CV system. Neuroimaging studies have shown that increased amygdala activity is associated with early markers of atherosclerosis, including carotid intima-media thickness, heightened blood pressure reactivity, enhanced inflammatory response to psychological stress, and increased all-cause mortality (29–31). Moreover, elevated resting amygdala activity in individuals without established CV disease has been linked to a higher incidence of future CV events. This association appears to be mediated by increased bone marrow activity and leukocyte mobilisation, which promote arterial inflammation and accelerate atherogenesis (32).

The Hypothalamus is located at the base of the diencephalon, flanking the third ventricle. It integrates limbic, cortical, and visceral inputs to coordinate autonomic and neuroendocrine output to the heart.

Within the hypothalamus, the paraventricular nucleus (PVN) has a central role in integrating afferent and efferent CV signals and modulating the baroreflex sensitivity and cardiovagal responses. The PVN is also a central component of the hypothalamic–pituitary–adrenal (HPA) axis. In response to stress, it releases corticotropin-releasing hormone (CRH), which stimulates the anterior pituitary gland to secrete adrenocorticotropic hormone (ACTH). ACTH, in turn, activates the adrenal cortex, leading to the release of cortisol, the primary stress hormone (33). While acute elevations in cortisol serve adaptive functions during stress, chronic hypercortisolaemia may contribute to both myocardial and cerebral injury (34). In addition to its well-established anti-inflammatory effects, evidence suggests that sustained cortisol exposure may exert paradoxical pro-inflammatory and pro-atherogenic effects, contributing to CAD, vascular dysfunction, and microglial dysregulation (35–37). Dysregulation of hypothalamic function may promote sustained sympathetic activation, impaired parasympathetic tone, and chronic neuroendocrine imbalance, thereby contributing to the development and progression of cardiovascular disease.

The Thalamus, below the cerebral cortex and above the brainstem, on either side of the third ventricle, is an integrative relay between peripheral cardiovascular signals and higher brain centres involved in autonomic, emotional, and cognitive regulation (38). Through its extensive connections with the brainstem, hypothalamus, and cerebral cortex, the thalamus receives afferent input related to heart rate, blood pressure, and visceral sensations and transmits these information to key regions of the CAN, including the insula and ACC. Thalamic neuronal activity is closely modulated by cardiac and respiratory rhythms, supporting the synchronisation of cardiovascular and respiratory function. By integrating sensory, autonomic, and limbic inputs, the thalamus contributes to the regulation of sympathetic and parasympathetic activity and links emotional and stress-related states to adaptive cardiovascular responses (39).

##### 2.1.1.3 Brainstem structures

The brainstem, comprising the midbrain, pons, and medulla oblongata, contains critical sensory and motor nuclei and serves as the principal integrative and regulatory centre of the heart–brain axis (2, 4, 21, 27, 40). It integrates peripheral cardiovascular signals with autonomic, emotional, and neurohumoral processes through specialised nuclei; the upper brainstem mediates responses to stress, pain, and arousal, whereas the lower brainstem governs circulation and respiration.

Afferent information from arterial baroreceptors, peripheral and central chemoreceptors, cardiac mechanoreceptors, and visceral pathways converges primarily in the nucleus tractus solitarius (NTS), located in the dorsomedial medulla. NTS is the primary sensory integration centre for CV signals processing haemodynamic and metabolic inputs to regulate heart rate, blood pressure, and ventilation, and coordinating the baroreflex and chemoreflex.

In the midbrain, the periaqueductal grey (PAG) integrates cortical, limbic, and cardiac nociceptive inputs and modulates cardiovascular responses during stress and pain. Increased PAG activity has been associated with reduced baroreflex sensitivity and enhanced sympathetic tone, thereby facilitating adaptive responses to cognitive and emotional challenges.

The medulla contains several key autonomic nuclei, including the vasomotor centre and rostral ventrolateral medulla (RVLM), which regulate sympathetic outflow and peripheral vascular resistance. It also contains the dorsal vagal complex, comprising the NTS, nucleus ambiguus, dorsal motor nucleus of the vagus, and area postrema. The nucleus ambiguus provides the main parasympathetic efferent control to the heart, contributing to cardiac rhythm stabilisation. The NTS receives input from cardiac and vascular chemoreceptors and mechanoreceptors, as well as pulmonary and central pathways, and relays this information to respiratory centres such as the pre- Bötzinger complex, considered a key respiratory rhythm generator, as well as to the parabrachial nucleus, and locus coeruleus**,** thereby promoting physiological coordinated cardiovascular and respiratory function.

Within the pons, the locus coeruleus represents the primary source of central noradrenaline. During states of arousal, increased noradrenergic activity enhances sympathetic tone and facilitates cognitive processing. Through its direct connections with the amygdala and hypothalamus, the locus coeruleus plays a central role in modulating stress responses.

Through its connections with the hypothalamus and limbic system, the brainstem also influences neuroendocrine and immune pathways, including the HPA axis and inflammatory signaling. Following neurological injury, dysregulation of these networks leads to sympathetic overactivation and catecholamine excess, predisposing patients to arrhythmias, stress-induced cardiomyopathy, and sudden cardiac death.

#### 2.1.2 Sympathetic and parasympathetic systems

The ANS, comprising sympathetic and parasympathetic branches, together with neuroendocrine mechanisms mediated by the HPA axis, collectively regulate heart rate, myocardial contractility, vascular resistance, and systemic haemodynamics (33, 41).

Forebrain regions project to autonomic nuclei within the medulla and spinal cord, forming part of the extrinsic cardiac nervous system. This system integrates sympathetic and parasympathetic inputs that regulate the intrinsic cardiac nervous system, composed of cardiac ganglia distributed within the atrial and ventricular walls (27). Sympathetic activation originates from neurons in the RVLM, which project to the preganglionic neurons located bilaterally in the intermediolateral column of the thoracic spinal cord. These preganglionic fibres synapse with postganglionic sympathetic neurons in the intrathoracic ganglia, including the stellate ganglion. Postganglionic fibres subsequently project to the heart, where they modulate sinoatrial and atrioventricular nodal activity, myocardial contractility, and coronary vasomotor tone (42).

Sympathetic activation also stimulates adrenal medullary release of adrenaline and noradrenaline, which regulate myocardial perfusion and vascular tone, and activates the renin-angiotensin-aldosterone system (RAAS), promoting vasoconstriction and sodium retention.

The ANS is closely integrated with the *immune system* (43). Autonomic fibres densely innervate lymphoid organs such as the spleen and lymph nodes, as well as hematopoietic stem cells within the bone marrow, all of which express *β*-adrenergic receptors (44). Through these pathways, sympathetic signaling modulates leukocyte mobilization, inflammatory responses, and vascular inflammation, linking neurogenic stress responses to atherosclerotic progression.

Parasympathetic innervation of the heart arises from preganglionic neurons in the nucleus ambiguus in the brainstem. These fibres travel along the vagus nerve (cranial nerve X), and synapse with postganglionic neurons in the ganglia of the intracardiac nervous system (45).

Sympathetic and parasympathetic efferent neurons converge within atrial and ventricular cardiac ganglia, where they interact to finely regulate cardiac electrical and mechanical activity. The dynamic balance between these two systems is essential for cardiovascular homeostasis, and its disruption contributes to arrhythmogenesis, myocardial dysfunction, and adverse cardiovascular outcomes.

#### 2.1.3 The intrinsic cardiac nervous system (ICNS)

Is organised into multiple interconnected ganglia, located within the epicardial fat surrounding the atria, atrioventricular junction, ventricles, the origin of coronary arteries, and pulmonary veins. These ganglia are linked by a dense network of nerve fibres forming intracardiac plexuses that innervate the myocardium, conduction system, and coronary vasculature (46).

The neuronal population of the ICNS includes efferent, afferent, and interneural elements. Efferent neurons mediate parasympathetic and sympathetic influences on cardiac tissues, regulating pacemaker function, conduction velocity, cardiomyocyte contractility, and myocardial perfusion. In contrast, afferent neurons convey sensory information related to mechanical, chemical, and metabolic stimuli (47, 48). The distribution of left- and right-sided sympathetic fibres over the ventricles is asymmetrical and exhibits considerable interindividual variability. In conditions of sympathetic overactivity, such asymmetrical distribution may lead to regional differences in action potential duration and myocardial repolarization, thereby creating an electrophysiological substrate for ventricular arrhythmias (49).

Structural and functional ICNS alterations are implicated in atrial and ventricular arrhythmias, heart failure, and sudden cardiac death. Abnormal ganglionic activity may promote electrical instability and autonomic imbalance, establishing the ICNS as a therapeutic target for neuromodulation and ganglionated plexus ablation (50).

### 2.2 Heart-to-brain pathways

Afferent pathways from the heart to the brain originate from chemoreceptors and baroreceptors in the atria, ventricles, the aortic arch, carotid sinus and coronary and pulmonary arteries (51). Signals from these receptors are transmitted through spinal nerves and cardiac vagal afferents to the NTS in the brainstem. The NTS serves as the primary sensory integration center for cardiovascular input, generating autonomic reflexes and relaying information to higher brain regions, including the locus coeruleus, thalamus, PAG, amygdala, and hypothalamus. These signals are subsequently projected to cortical structures such as the insular cortex and ACC, enabling integrated hemodynamic regulation through descending efferent pathways (40).

Emerging evidence also suggests that the brain can both perceive and influence the progression of atherosclerotic lesions. The adventitia of atherosclerotic arteries is innervated by sensory and sympathetic fibres and contains clusters of immune cells, forming the *neuroimmune CV interfaces* (52). The development of atherosclerotic plaques has been associated with activation of hypothalamic nuclei involved in sympathetic regulation, supporting a role for central neural mechanisms in modulating plaque progression and vascular inflammation (53).

### 2.3 Mechanisms of heart-brain interactions

#### 2.3.1 Neuroinflammation and systemic immune response

**Figure 2:**
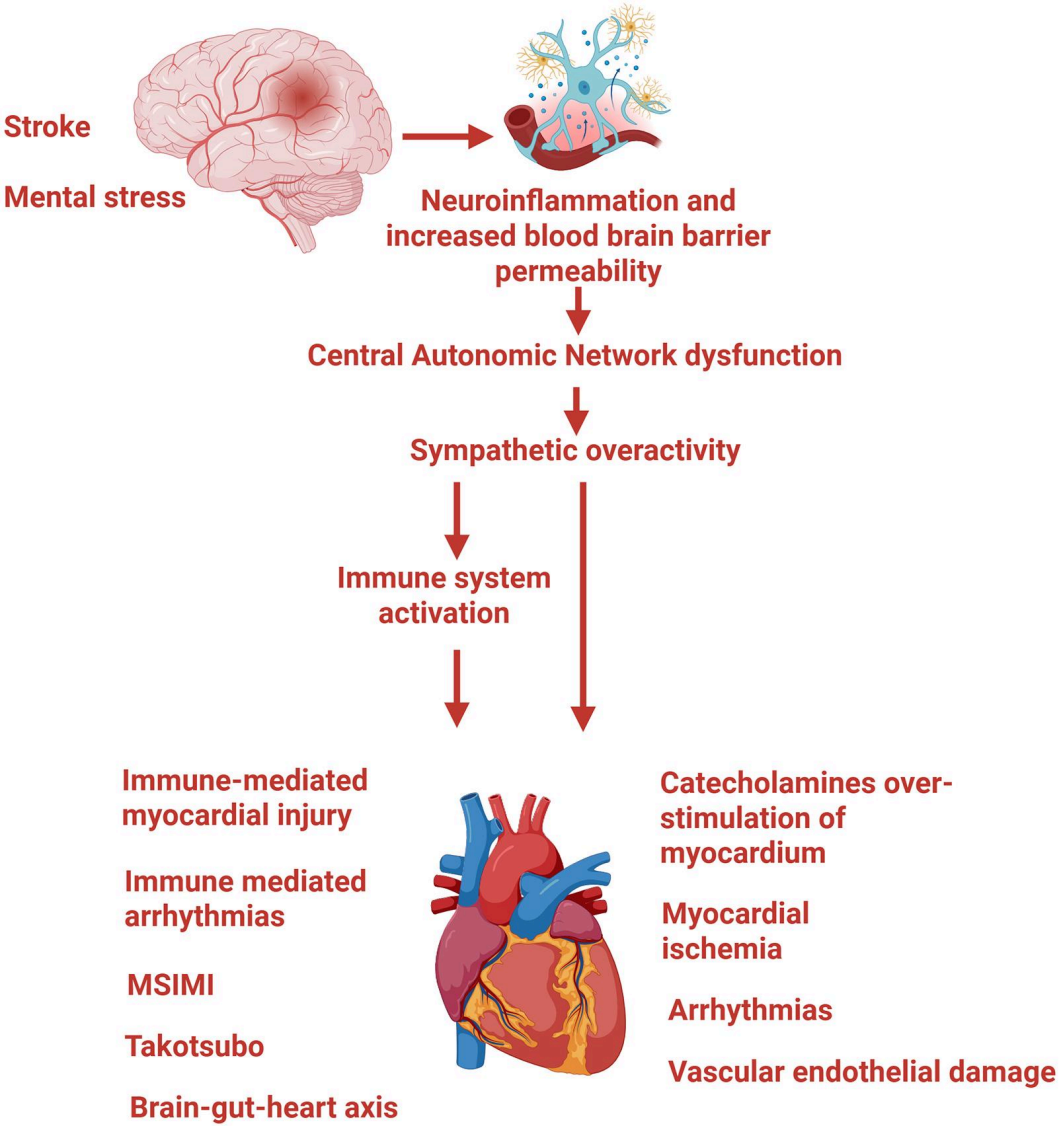
Effects of cerebral ischaemia on the heart. Stroke and mental stress initiate neuroinflammation and increased BBB permeability, disrupting the neurovascular unit and impairing the brain's ability to autoregulate vascular tone. The resulting structural and functional dysregulation of the CAN leads to sustained sympathetic overactivity, which propagates cardiac injury through two converging pathways. Through immune system activation, sympathetic overdrive promotes mobilisation of monocytes, neutrophils, and haematopoietic stem cells, generating a systemic pro-inflammatory state. This drives immune-mediated myocardial injury, including macrophage infiltration, fibrotic remodelling, and coronary microvascular dysfunction, as well as immune-mediated arrhythmias, MSIMI, Takotsubo cardiomyopathy, and gut-brain-heart axis dysregulation through increased gut permeability and translocation of pro-inflammatory microbial products. Through direct catecholaminergic overstimulation of the myocardium, excessive catecholamine release activates *β*-adrenergic receptors, increasing myocardial oxygen demand and promoting calcium overload, mitochondrial dysfunction, and contraction band necrosis. This pathway contributes to myocardial ischaemia, arrhythmias, including neurogenic atrial fibrillation and ventricular tachyarrhythmias, and vascular endothelial damage through platelet activation and impaired microvascular function. BBB, blood brain barrier; CAN, Central autonomic network; MSIMI, mental stress-induced myocardial ischaemia.

Both ischemic and hemorrhagic stroke induce neuronal cells necrosis, leading to increased blood-brain barrier (BBB) permeability and disruption of the structure and function of the neurovascular unit (NVU) (3, 54). This impairment compromises the brain's ability to autoregulate vascular tone, a phenomenon referred to as neurovascular uncoupling (Figure 2).

Cerebral ischemia activates microglia and astrocytes in response to damage-associated molecular patters (DAMP), released by necrotic cells. While this inflammatory response initially serves a protective function, aiming at limiting further injury, prolonged or severe ischemia may exceed the compensatory capacity of glial cells, resulting in secondary cellular damage and progressive neurological dysfunction.

Under pathological conditions, glial cells may shift from an anti-inflammatory, reparative (M2) phenotype, to a pro-inflammatory (M1) phenotype. This phenotipic transition promotes the release of pro-inflammatory cytokines and activation of cerebral endothelial cells, further compromising BBB integrity (55–58). Over time, sustained neuroinflammation contributes to structural and functional dysregulation of the central autonomic network (CAN) (59). These alterations may result in increased sympathetic tone and/or withdrawal of parasympathetic tone, impairing autonomic control of the heart, and activating the HPA axis (58, 60).

Persistent neuroinflammation may also promote the leakage of pro-inflammatory cytokines into the systemic circulation, transforming a localised central nervous system response into a generalised systemic inflammatory state (61, 62). Although these processes are most prominent in the acute phase of stroke, they may persist for months, contributing to long-term cardiovascular and neurological complications.

#### 2.3.2 Direct catecholamine-mediated myocardial overstimulation

The autonomic and inflammatory consequences of neuroinflammation converge on the myocardium through a second, overlapping mechanism: direct catecholamine-mediated cardiac injury. Prolonged sympathetic overactivity may result in excessive catecholaminergic stimulation of the myocardium, leading to intracellular calcium overload in cardiomyocytes, mitochondrial dysfunction, contraction band necrosis, and coronary microvascular endothelial impairment (60, 63). Activation of *β*-adrenergic receptors by catecholamines enhances myocardial contractility and heart rate, thereby increasing myocardial oxygen demand. When oxygen demand exceeds supply, particularly in the presence of subclinical CAD, coronary demand ischemia may occur, potentially resulting in type 2 myocardial infarction (64, 65).

Sympathetic overdrive may destabilize atherosclerotic plaques, making them more prone to rupture. Excessive catecholamine release from sympathetic nerve endings also promotes inflammatory cell infiltration and fibrotic remodelling around the ganglionated plexi in the left atrium and pulmonary veins, creating a substrate for neurogenic atrial fibrillation (66, 67).

In addition, heightened sympathetic activity can induce life-threatening ventricular tachyarrhythmias, particularly in patients with inherited arrhythmogenic disorders such as long QT syndrome and catecholaminergic polymorphic ventricular tachycardia, which are characterised by myocardial ion channel dysfunction (68).

#### 2.3.3 Sympathetic effects on the immune system

Beyond its direct toxic effects on cardiomyocytes, sympathetic overactivation also shapes the inflammatory environment through its influence on immune cell activity. Increased sympathetic tone promotes the mobilisation of monocytes and neutrophils from the spleen and stimulates the proliferation of hematopoietic stem cells in the bone marrow, raising circulating leukocytes levels (69–71). These immune cells play a central role in the pathogenesis of atherosclerosis, MI, HF, and arrhythmias (43, 72–75).

Under physiological conditions, parasympathetic (vagal) activity exerts an anti-inflammatory effect. Acetylcholine, released from vagal efferent fibres inhibits the production of pro-inflammatory cytokines, including IL-1, IL-2, IL-6, and tumour necrosis factor-alpha (TNF-α), by tissue macrophages. This neuroimmune mechanism, known as the *cholinergic inflammatory reflex*, has a critical role in regulating immune and inflammatory responses (76, 77).

In the context of stroke, autonomic imbalance, characterised by increased sympathetic activity and reduced parasympathetic tone, impairs this protective reflex. The resulting loss of cholinergic inhibition allows inflammatory cytokine production to persist and amplify, sustaining systemic inflammation (78).

#### 2.3.4 Immune-mediated myocardial injury

The systemic inflammatory state generated through these neuro-immune pathways exerts direct pathological effects on the heart, through immune-mediated mechanisms that compromise myocardial structure and function. The normal heart contains a large population of resident macrophages distributed around the coronary arteries, within the endothelium, in the pericardial space, and near the conduction system. These resident immune cells interact closely with the cardiomyocytes, fibroblast and endothelial cells to maintain myocardial homeostasis (73, 79).

In conditions of chronic systemic inflammation, circulating cytokines activate tissue-resident cardiac macrophages, inducing a pro-inflammatory phenotype. Once activated, macrophages infiltrate the myocardium and stimulate microvascular endothelial cells, contributing to coronary microvascular dysfunction. This process may impair myocardial perfusion, promote LV dysfunction and the development of arrhythmias (80–82).

Moreover, pro-inflammatory cytokines drive conversion of cardiac fibroblast into myofibroblast, promoting extracellular matrix deposition and myocardial fibrosis (83, 84). Fibrotic remodeling alters electrical conduction and mechanical function, creating a substrate for arrhythmogenesis and progressive HF.

Cardiac macrophages also play a key role in the destabilisation of atherosclerotic plaques. They migrate into plaques and secrete proteolytic enzymes that degrade the fibrous cap, thereby increasing the risk of rupture and subsequent acute coronary events (85).

#### 2.3.5 Immune-mediated arrhythmias

In addition to the structural and perfusion consequences described above, immune activation has direct electrophysiological effects, establishing a clear mechanistic link between inflammation and cardiac rhythm disturbances. Several studies have demonstrated a direct relationship between elevated pro-inflammatory cytokines in the heart and the development of rhythm disorders, particularly AF (86–88). The myocardium and the conduction system contain a substantial population of leukocytes, primarily macrophages, which are electrically coupled to cardiomyocytes through gap junctions. These macrophages depolarise in synchrony with adjacent cardiomyocytes, thereby modulating membrane potentials and contributing to normal electrophysiological function (73, 89, 90).

Systemic immune activation, such as that occurring after stroke, can disrupt this finely regulated cellular communication, increasing the risk of arrhythmias. Immune response may also generate autoantibodies that interfere with cardiac ion channels, further destabilising electrical activity (91, 92). Atrial remodelling caused by myocardial fibrosis further promotes AF by inducing slowed and heterogeneous conduction within the atrial myocardium. Notably, the relationship between inflammation and AF is bidirectional: AF itself sustains and amplifies inflammation, which in turn accelerates structural and electrical remodelling, creating a self-perpetuating arrhythmogenic substrate (88).

#### 2.3.6 Vascular endothelial damage

Systemic inflammation and heightened sympathetic activity contribute to vascular endothelial injury by promoting platelet activation and leukocyte recruitment to the endothelial surface. These interactions induce a prothrombotic state, enhancing adhesion of leukocytes and platelets to the vessel wall and facilitating the development of arterial thrombus (93).

At the microvascular level, circulating pro-inflammatory cytokines directly impair endothelial function, leading to coronary microvascular dysfunction (94, 95). Endothelial injury following stroke has also been associated with reduced levels of endothelial micro-RNA-126, a short sequence of noncoding RNA which regulate vascular integrity and angiogenesis (96).

#### 2.3.7 Brain-gut-heart axis

A further dimension of heart-brain crosstalk involves the gastrointestinal tract. Through the brain-gut-heart axis, autonomic dysfunction and systemic inflammation trigger a cascade of gut-derived signals that amplify cardiovascular and neurological injury. Sympathetic overactivation and systemic inflammation can impair gastrointestinal function, leading to intestinal paralysis and increased gut permeability. These changes allow the translocation of bacteria and microbiota-derived endotoxins into the bloodstream (94, 97–99).

The entry of these microbial products into the circulation amplifies inflammatory cytokine levels, perpetuating systemic inflammation. The gut microbiota also contribute to inflammation metabolising dietary components such as choline, lecithin, and L-carnitine into trimethylamine-N-oxide (TMAO), a pro-atherogenic metabolite that activates macrophages and platelets, increasing the risk of thrombosis (100, 101). Systemic inflammation driven by gut-derived pathogens may compromise BBB integrity, exacerbating neuroinflammation and contributing to neural injury and dysfunction (102).

### 2.4 Clinical conditions of the heart-brain interaction

#### 2.4.1 Clinical impact of stroke on the heart

Cardiac complications occur in approximately 20% of patients following acute stroke, and account for nearly 20% of deaths within the first three months. They represent the second leading cause of mortality after neurologic complications [Table 1, (60, 103, 104)]. Cardiac abnormalities typically peak during the early days after stroke; approximately one-third can occur within four weeks and one-fifth within three months (103, 105).

**Table 1:** Incidence of neurogenic heart disease.

Clinical manifestations	Incidence
Myocardial injury: elevated hs-cTn	30–60% (118)
Chronic myocardial injury: persistently elevated hs-cTn, without clinical evidence of MI	11–40% (119, 312)
Acute myocardial injury: elevated cTn with a rising and/or falling pattern >20%, without clinical evidence of MI	15–25% (119, 312)
LV dysfunction: ejection fraction <50% or clinical HF	8–30% (16, 132, 134)
Acute myocardial infarction	∼1.6% (313, 314)
	∼50% in stroke patients with very high hs-cTn (121)
Concomitant acute ischemic stroke and STEMI	0.3–15% (116, 315)
ECG changes: QT interval prolongation, ST segment elevation or depression, deeply inverted T waves, atrio-ventricular and bundle branch blocks	20–65% (94, 146, 148)
Post-stroke AF: patients without known AF	24% on long-term Monitoring (16)
MSIMI:	20–70% of patients with stable CAD (166, 169)
Takotsubo cardiomyopathy:	∼1% (176, 177)

hs-cTn, high-sensitivity cardiac troponin; MI, myocardial infarction; HF, heart failure; AF, atrial fibrillation; MSIMI, mental stress-induced myocardial ischemia; CAD, coronary artery disease; STEMI, ST-segment elevation myocardial infarction.

Stroke heart syndrome (SHS) is defined as the development of new CV complications or the exacerbation of pre-existing CAD, within 30 days of an acute ischemic stroke or other acute neurological conditions, including hemorrhagic stroke, subarachnoid hemorrhage, traumatic brain injury, and seizures (16, 65, 105, 106).

SHS encompasses a broad spectrum of cardiac manifestations, including myocardial injury, acute MI, LV systolic dysfunction, HF, and stress-induced cardiomyopathy (Takotsubo syndrome). Additional manifestations include electrocardiographic (ECG) abnormalities, atrial and ventricular arrhythmias (including AF) and sudden cardiac death [(107), Figure 2].

Although SHS may occur in individuals without previous cardiac disease, the severity of cardiac involvement is influenced by the presence of CV risk factors, advanced age, and underlying CAD (108, 109). Notably, more than half of patients with acute ischemic stroke and no history of cardiac disease, show evidence of subclinical coronary atherosclerosis, and approximately one-third have coronary stenosis >50% (110, 111).

The extent of cardiac involvement correlates with the severity and anatomical location of cerebral ischaemia, particularly in cases involving the insular cortex, underscoring the role of autonomic dysregulation in the pathogenesis of SHS (112–114). The clinical presentation varies widely, ranging from asymptomatic laboratory or ECG abnormalities to severe conditions requiring immediate intervention.

**Figure 3:**
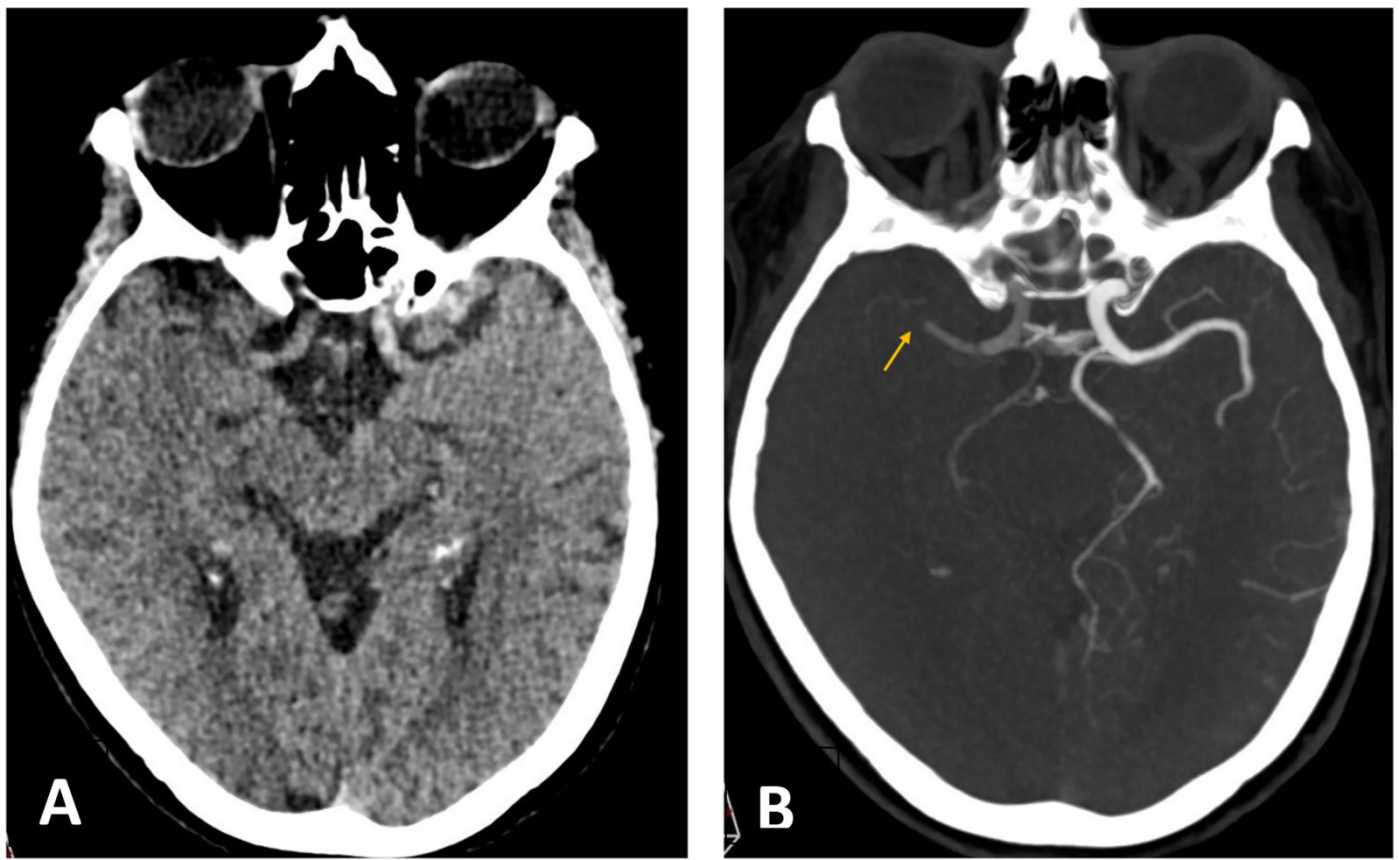
A 76-year-old woman was admitted to hospital with a right hemispheric stroke. Her National Institutes of Health Stroke Scale (NIHSS) score was 18. Non-contrast CT **(A)** showed no signs of established cerebral infarction. CT angiography **(B)** revealed an occlusion of the M1 segment of the right middle cerebral artery (MCA) (arrow).

**Figure 4:**
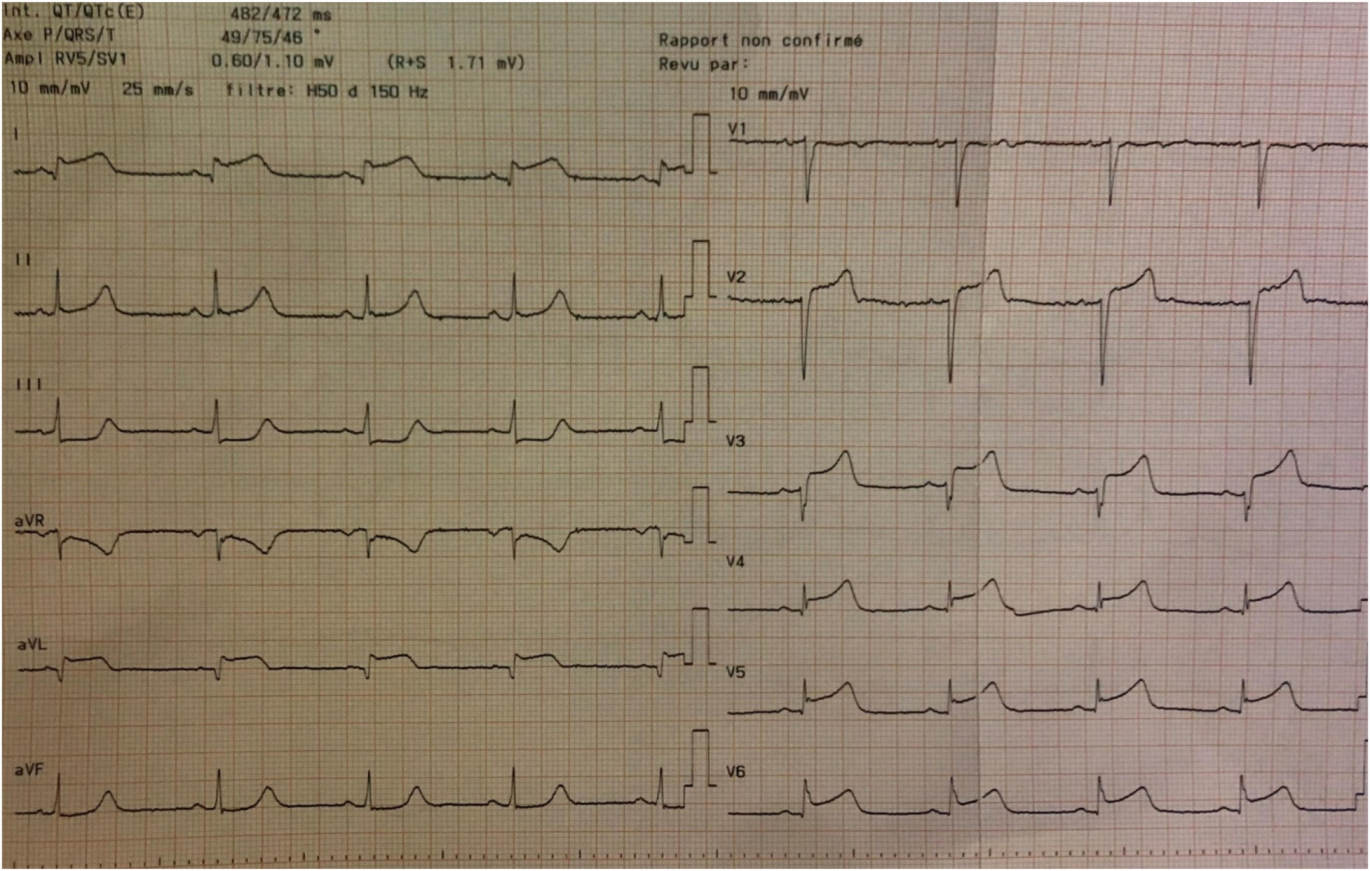
An acute myocardial infarction is also detected, which occurred without symptoms because of the unconscious state induced by the stroke. The ECG is consistent with a ST-segment elevation myocardial infarction (STEMI). Troponin 85 ng/L. Transthoracic echocardiogram in the emergency department demonstrated a reduced left ventricular ejection fraction (LVEF) of 30% and apical and septal hypokinesia, and no evidence of intracardiac thrombus. Urgent cerebral artery thrombectomy is planned, immediately followed by coronary angiography and myocardial revascularisation.

**Figure 5:**
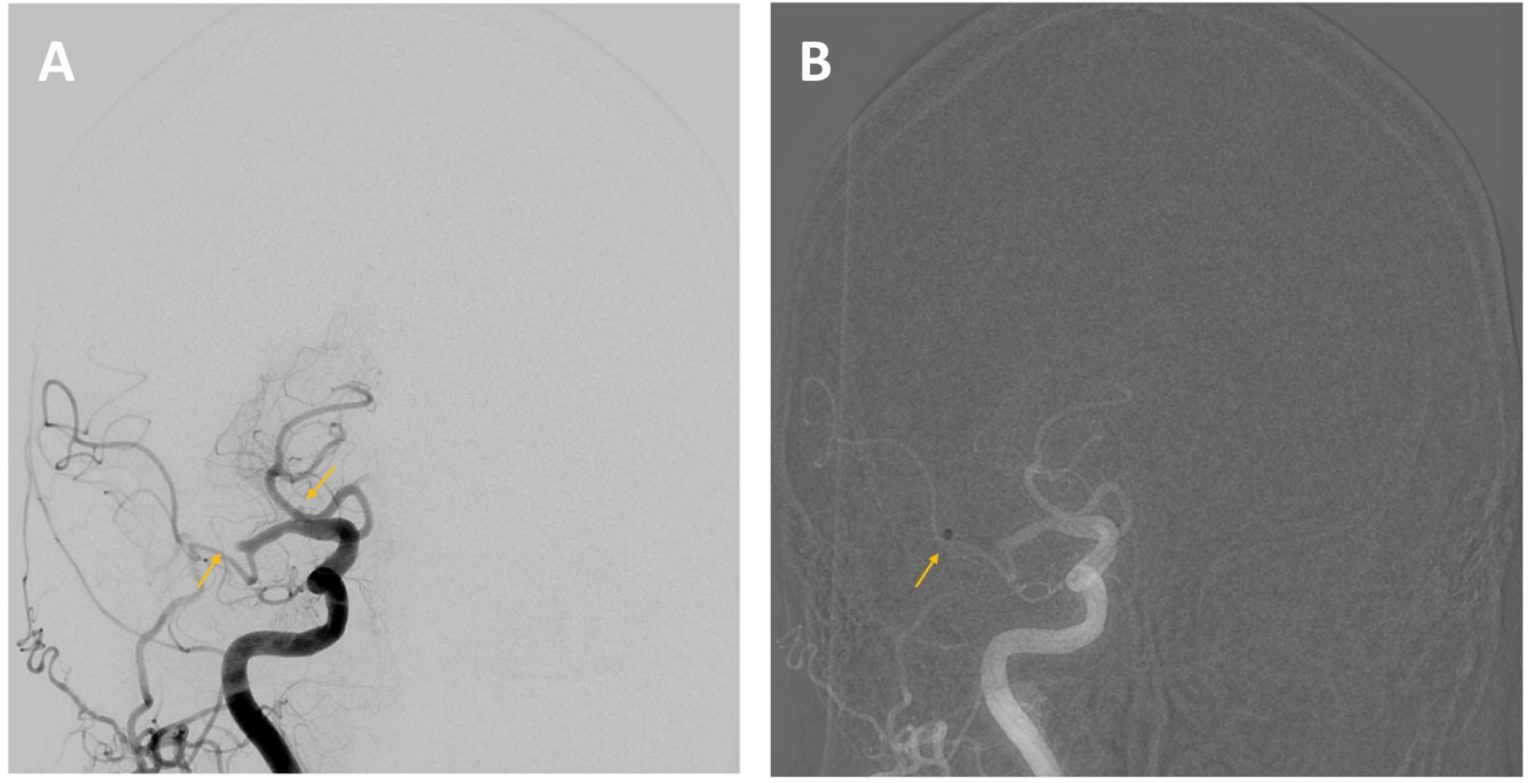
**(A)** An angiographic run of the right carotid axis confirmed occlusion of the M1 segment of the right middle cerebral artery (MCA) and demonstrated an additional occlusion at the origin of the A1 segment of the anterior cerebral artery (ACA). **(B)** Mechanical thrombectomy was performed using a contact aspiration catheter (the radiopaque catheter tip is indicated by the yellow arrow), resulting in successful reperfusion with a Thrombolysis in Cerebral Infarction (TICI) grade of 2c, indicating near-complete restoration of distal blood flow with minimal residual occlusion.

**Figure 6:**
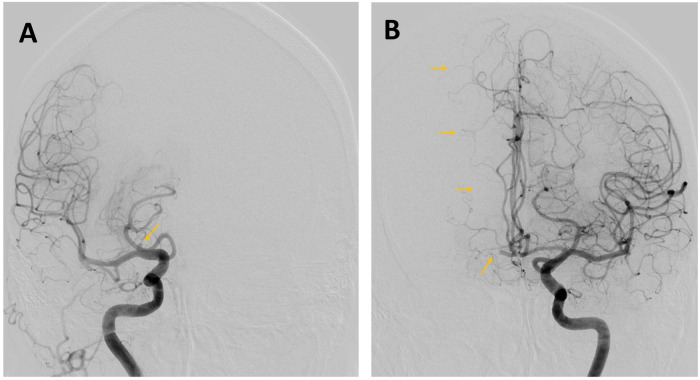
Results of the thrombectomy. **(A)** An angiographic run of the right carotid axis shows complete revascularisation of the right MCA territory, with persistent occlusion at the origin of the A1 segment of the right ACA (arrow). **(B)** An angiographic run of the left carotid axis demonstrates contralateral compensation of the ACA territory via the anterior communicating artery (arrow).

**Figure 7:**
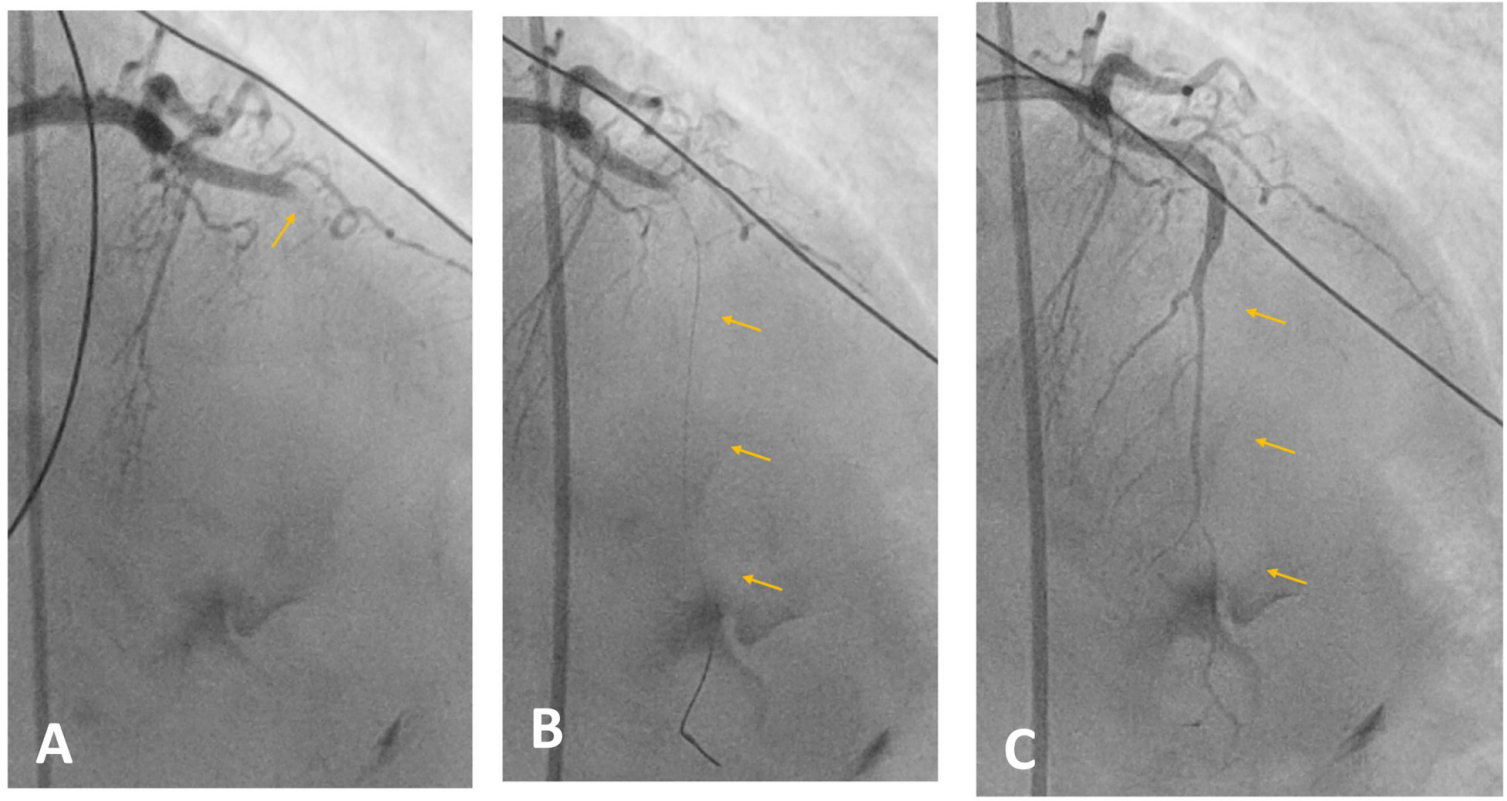
Coronary angiography. **(A)** Occlusion of the left anterior descending artery due to an intraluminal thrombus. **(B)** Mechanical thrombectomy with successful recanalisation of the artery. **(C)** Final angiographic result showing restored coronary flow.

**Figure 8:**
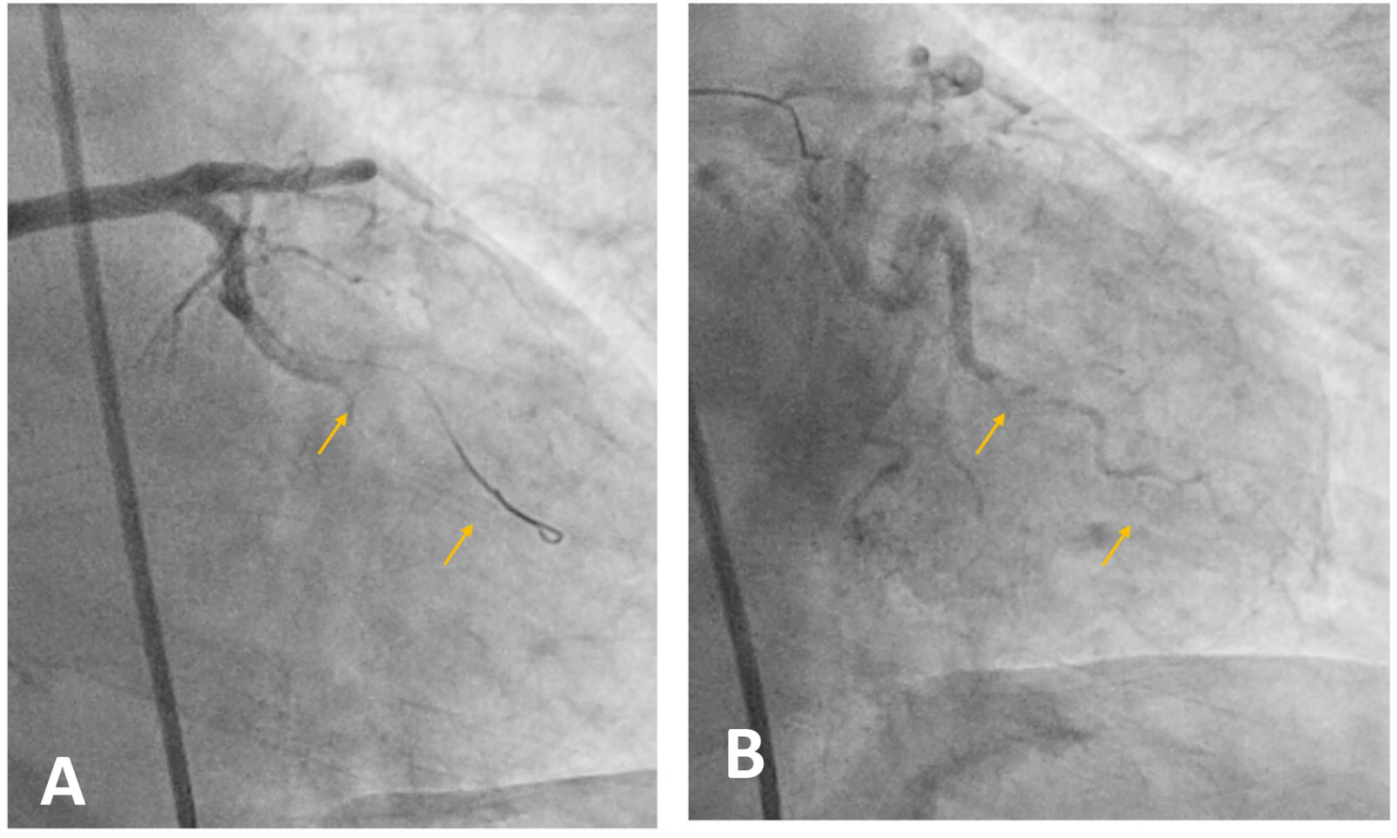
Coronary angiography. **(A)** The first marginal branch shows a short acute occlusion (<10 mm), classified as type B1, with an intraluminal thrombus at this site. **(B)** Recanalisation of the artery by thrombectomy followed by coronary stent placement.

Acute ischemic stroke may occur simultaneously with MI [(115), Figures 3–8]. Although this presentation is uncommon, it is associated with a high in-hospital mortality rate, approaching 25% (116, 117). In such cases, the clinical presentation of MI may be atypical, particularly in older patients with neurological deficits, such as impaired consciousness or aphasia, which may obscure classical ischemic symptoms.

##### 2.4.1.1 Myocardial injury

**Figure 9:**
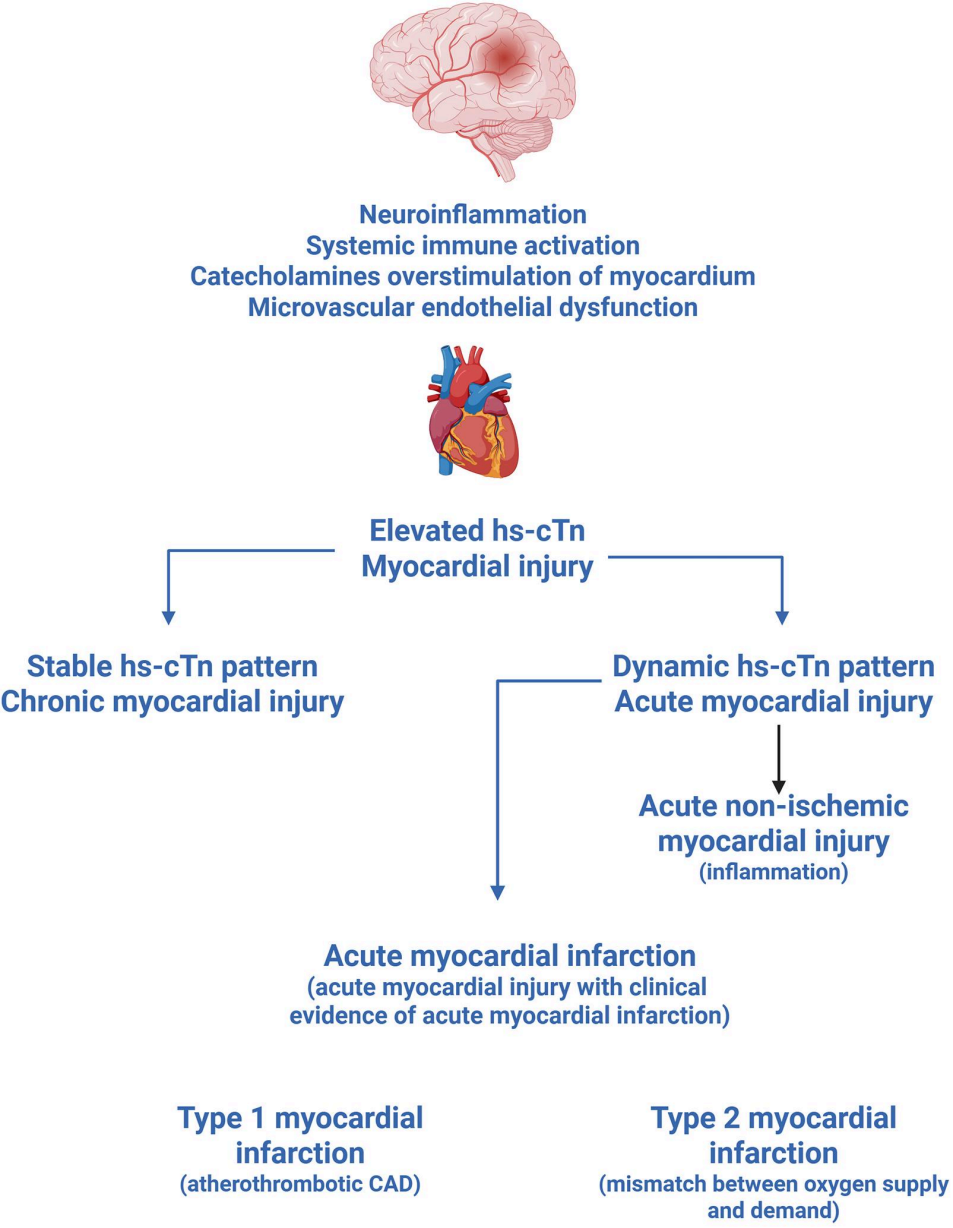
Neurogenic myocardial injury following acute ischaemic stroke. Stroke-induced neuroinflammation, systemic immune activation, catecholamine overstimulation of the myocardium, and microvascular endothelial dysfunction all contribute to cardiomyocyte injury, reflected by elevated hs-cTn levels. Serial troponin measurements are essential to classify the pattern of injury. A stable hs-cTn pattern indicates chronic myocardial injury, characterised by persistently elevated or minimally fluctuating levels. A dynamic hs-cTn pattern, defined by a rise and/or fall exceeding 20%, indicates acute myocardial injury, which may represent either acute non-ischaemic injury driven by inflammation, or progression to acute MI when accompanied by clinical evidence of myocardial ischaemia. Approximately 50% of stroke patients with markedly elevated hs-cTn fulfil criteria for MI. When MI criteria are met, two subtypes are distinguished: Type 1 MI, caused by atherothrombotic coronary artery disease, and Type 2 MI, resulting from an oxygen supply-demand mismatch in the context of tachyarrhythmias, hypertensive emergencies, or respiratory failure. hs-cTn, high-sensitivity cardiac troponin; CAD, coronary artery disease; MI, myocardial infarction.

More than 50% of patients with acute ischemic stroke show evidence of myocardial injury, defined by elevated high-sensitive cardiac troponin (hs-cTn) levels in the absence of clinical features of myocardial infarction (MI) (16). These troponin elevations are typically transient and resolve within a few days. Serial troponin measurements are essential to distinguish *chronic myocardial injury*, characterised by persistently elevated or minimally fluctuating troponin levels, from *acute myocardial injury*, which is defined by a dynamic change exceeding 20% [(118, 119), Figure 9]. Acute myocardial injury may represent an early stage in the progression towards MI, which is defined as myocardial injury with clinical evidence of acute myocardial ischemia.

Approximately 50% of patients with markedly elevated hs-cTn, either exceeding the diagnostic threshold for non-ST-segment-elevation MI (NSTEMI) or showing a significant dynamic rise and/or fall, may fulfil criteria for MI (120–122).

A substantial proportion of stroke patients with MI may have type 2 MI, resulting from an imbalance between myocardial oxygen supply and demand, often in the context of tachyarrhythmias, hypertensive emergencies, or respiratory failure (123).

Elevated cTn levels in patients with stroke, including those with minor stroke and transient ischemic attack (TIA), are strongly associated with an increased risk of cognitive impairment, recurrent vascular events, and both short and long-term mortality (110, 118, 124, 125). In a prospective cohort study, the long-term risk of major cardiovascular events (MACE) following stroke or (TIA) was approximately 13% within 1 year after discharge and 35% after a median follow-up of 4.4 years (126).

Brain natriuretic peptide (BNP) levels are frequently elevated during the first days following stroke and are associated with a higher risk of paroxysmal AF, worse functional outcomes, and increased mortality (127–129). BNP concentrations typically decline after the acute phase.

Current stroke management guidelines recommend early measurement of cardiac troponin to identify patients at high risk of adverse outcomes; however, they provide limited guidance regarding the subsequent diagnostic evaluation and management strategy (130). The coexistence of acute stroke and MI, especially ST-segment elevation MI (STEMI) poses significant diagnostic and therapeutic challenges due to narrow therapeutic windows and competing treatment priorities (131).

##### 2.4.1.2 LV dysfunction and heart failure

Reduced LV ejection fraction (EF <50%), or clinically significant HF, accompanied by elevated BNP levels, may occur in approximately one-third of patients within the first few days after a stroke, and is associated with unfavourable functional outcomes (16, 132–134).

Systemic inflammation and immune activation may contribute to the development or progression of HF by exacerbating neurohumoral and autonomic dysregulation (135). In particular, brain injury involving the right insular cortex has been associated with impaired global LV longitudinal strain, even in patients without previous cardiac dysfunction (136). These cardiac abnormalities are associated with worse prognosis due to hemodynamic impairment and reduced systemic perfusion. However, since LV function prior to strokes is often unknown, accurately estimating the true prevalence of post-stroke cardiac dysfunction remains uncertain.

##### 2.4.1.3 Arrhythmias

Continuous cardiac monitoring during the acute phase of stroke reveals that approximately 25% of patients experience clinically relevant arrhythmias, predominantly tachyarrhythmias (137). AF identified first identified after a stroke is termed AF detected after stroke (AFDAS) (65, 138, 139). AFDAS may arise from a combination of neurogenic mechanisms, secondary to acute cerebral injury (neurogenic AFDAS), and underlying atrial vulnerability (cardiogenic AFDAS). It is frequently associated with strokes involving the insular cortex and other limbic structures that regulate autonomic control of cardiac rhythm (140, 141). Emerging evidence suggests that AFDAS may represent a distinct clinical entity compared with previously diagnosed AF and may be associated with a lower risk of stroke recurrence (142, 143). Regardless of the differences, current recommendations support the initiation of oral anticoagulation therapy in all patients with AFDAS for secondary stroke prevention (144).

Electrocardiographic (ECG) abnormalities, including QT interval prolongation, ST segment elevation or depression, deeply inverted T waves (so called “cerebral T waves”), and atrio-ventricular or bundle branch blocks, are frequently observed during the first days following a stroke (145). These changes are often transient and are particularly common in patients with elevated cTn or pre-existing heart disease. However, their presence has been associated with poorer clinical outcomes (146–148).

#### 2.4.2 Clinical impact of psychological stress on the heart

Acute and chronic psychological stress can trigger a wide range of CV abnormalities (149). Perceived threats are processed by the CNS, which evaluates environmental stimuli and initiates physiological responses aimed at adaptation. Stress arises when psychological demands exceed an individual's capacity to make adaptive changes (150). In such circumstances, the brain activates hemodynamic, neuroendocrine, and immune changes through mechanisms that partially overlap with those observed in ischemic brain injury. Elevated levels of stress-induced noradrenaline may further influence microglial function via *β*-adrenergic receptors, thereby promoting neuroinflammatory signalling (151).

*Acute mental stress* refers to short-term exposure to intense stressors such as anger, fear, acute occupational strain, natural disasters, or emotionally charged sporting events (152–154). Epidemiological studies have also documented increased rates of cardiovascular events on the first workday of the week and during the Christmas and New Year holiday period (155, 156).

*Chronic psychological stress* involves prolonged or repetitive exposure to stress, as occurs in conditions such as depression and anxiety (157, 158). Unlike acute stress responses, chronic mental stress is characterized by sustained sympathetic activation and systemic low-grade inflammation. These processes contribute to the progression of atherosclerosis and destabilization of coronary plaque, thereby increasing the risk of myocardial ischemia (159, 160).

##### 2.4.2.1 Three major clinical entities are recognised

Mental stress-induced myocardial ischemia (MSIMI), is defined as transient myocardial ischemic response triggered by psychological stress rather than physical exertion (5, 161). In addition to MSIMI, ischemia with non-obstructive coronary arteries (INOCA), and myocardial infarction with non-obstructive coronary arteries (MINOCA), represent distinct but partially overlapping clinical entities characterised by complex interactions between central neural regulation and coronary pathophysiology.

INOCA refers to chronic or recurrent myocardial ischemia in patients with angiographically non-obstructive (<50%) coronary arteries and is primarily driven by coronary microvascular dysfunction, impaired vasodilatory reserve, and epicardial or microvascular vasospasm, irrespective of the precipitating trigger (162). In contrast, MINOCA denotes an acute myocardial infarction that fulfils universal diagnostic criteria in the absence of obstructive coronary stenoses (163).

MSIMI may manifest as angina, MI, arrhythmias, LV dysfunction, and stress-induced cardiomyopathy (Takotsubo syndrome) (155, 164–166). Its occurrence is influenced by an individual's CV risk profile (167). MSIMI is associated with a twofold increase in the incidence of major adverse cardiac events (MACE), even after adjustment for established CV risk factors (168). Among patients with stable CAD, the reported prevalence of MSIMI ranges from 20% to 70%, reflecting variability in the type of psychological stress applied, diagnostic methodologies used, and patient psychological characteristics (169). In these patients, ischemic responses may be triggered not only by acute emotional stress but also by everyday environmental stressors (170).

MSIMI may occur more frequently than exercise-induced myocardial ischemia and can be present in individuals who do not exhibit ischemia during exercise testing. Furthermore, it is not consistently associated with the angiographic severity of CAD (171). Angiographic and physiological studies suggest that MSIMI is largely related to impaired coronary microcirculatory function secondary to endothelial dysfunction (172, 173). MSIMI is often under-recognised because it may occur in the absence of chest pain. Holter monitoring studies have shown that more than two-thirds of ischemic episodes occur during daily activities, without anginal symptoms (174). Moreover, strong evidence indicates that mental stress–induced myocardial ischemia is independently associated with an increased risk of future cardiac events and mortality in patients with stable CAD (166).

Stress cardiomyopathy (Takotsubo syndrome, TTS) typically presents as congestive HF, clinically mimics STEMI. It is usually triggered by intense emotional or physical stress (175). The condition is characterised by reversible balloon-like LV wall motion abnormalities, most commonly involving the apical segments, although mid-ventricular or focal patterns may also occur, in the absence of significant coronary artery obstruction (65). In the context of acute stroke, this condition is often referred to as “neurogenic stress cardiomyopathy” emphasizing the neurological trigger underlying the cardiac dysfunction.

TTS accounts for approximately 1%–3% of patients initially presenting with suspected STEMI, and occurs in about 1% of patients with acute stroke (176, 177). It predominantly affects postmenopausal women, likely due to reduced oestrogen levels which may enhance sympathetic vasoconstriction and impair endothelial function. TTS may also be triggered by intense, positive emotional experiences, a phenomenon described as “Happy heart syndrome” (178). Although LV systolic function typically recovers over time, long-term prognosis is comparable to that of patients with myocardial infarction (179). Contrary to earlier assumptions that TTS is a transient and benign condition, it is now recognized to carry substantial rates of acute complications, similar to those observed in acute coronary syndromes (179).

The underlying pathophysiology is thought to involve overstimulation of the sympathetic nervous system, in response to acute stress. Histopathological findings are consistent with catecholamine-mediated myocardial toxicity and include focal infiltration of mononuclear inflammatory cells, increased extracellular matrix protein deposition, and contraction band necrosis (myocytolysis).

Neuroimaging studies have shown structural alterations in limbic regions and impaired functional connectivity within the cortico-limbic network in patients with TTS (180). Affected regions include the prefrontal cortex, right insula, amygdala, hypothalamus, cingulate cortex, and thalamus, all of which regulate the autonomic function and cardiovascular responses to stress. Abnormal functional connectivity patterns may persist for weeks after the acute cardiac event, even following recovery of LV function (181). Furthermore, reduced functional brain connectivity may precede the development of TTS by several years. Retrospective analyses have shown increased amygdalar activity in individuals who later developed TTS, suggesting that heightened limbic reactivity may predispose susceptible individuals by amplifying autonomic and neurohormonal responses to future stressors (182–184).

##### 2.4.2.2 Stress-induced arrythmias

Both acute and chronic stress increase the risk of arrhythmias and sudden cardiac death (SCD). The incidence of ventricular arrhythmia is fivefold higher during periods of intense anger compared to control periods (185, 186). Moreover, anger and negative emotions are associated with the onset and progression of AF, even after adjustment for traditional CV risk factors and previous CAD (187). Negative emotional states activate the sympathetic nervous system while suppressing parasympathetic output (188). Catecholamine excess may increase the risk of arrhythmias by inducing electrical heterogeneity of ventricular depolarisation and repolarisation (189, 190). Another proposed mechanism is the “brain-heart laterality”, which suggests that lateralised imbalances in autonomic neural control may result in asymmetrical sympathetic stimulation of the heart, thereby increasing the risk of arrhythmias (191–193).

Conversely, positive emotional states have been shown to decrease CV reactivity to stress, reducing heart rate and blood pressure responses, arrhythmia development, thrombogenesis, and the risk of CAD (194).

#### 2.4.3 Clinical impact of cardiac disorders on the brain

**Figure 10:**
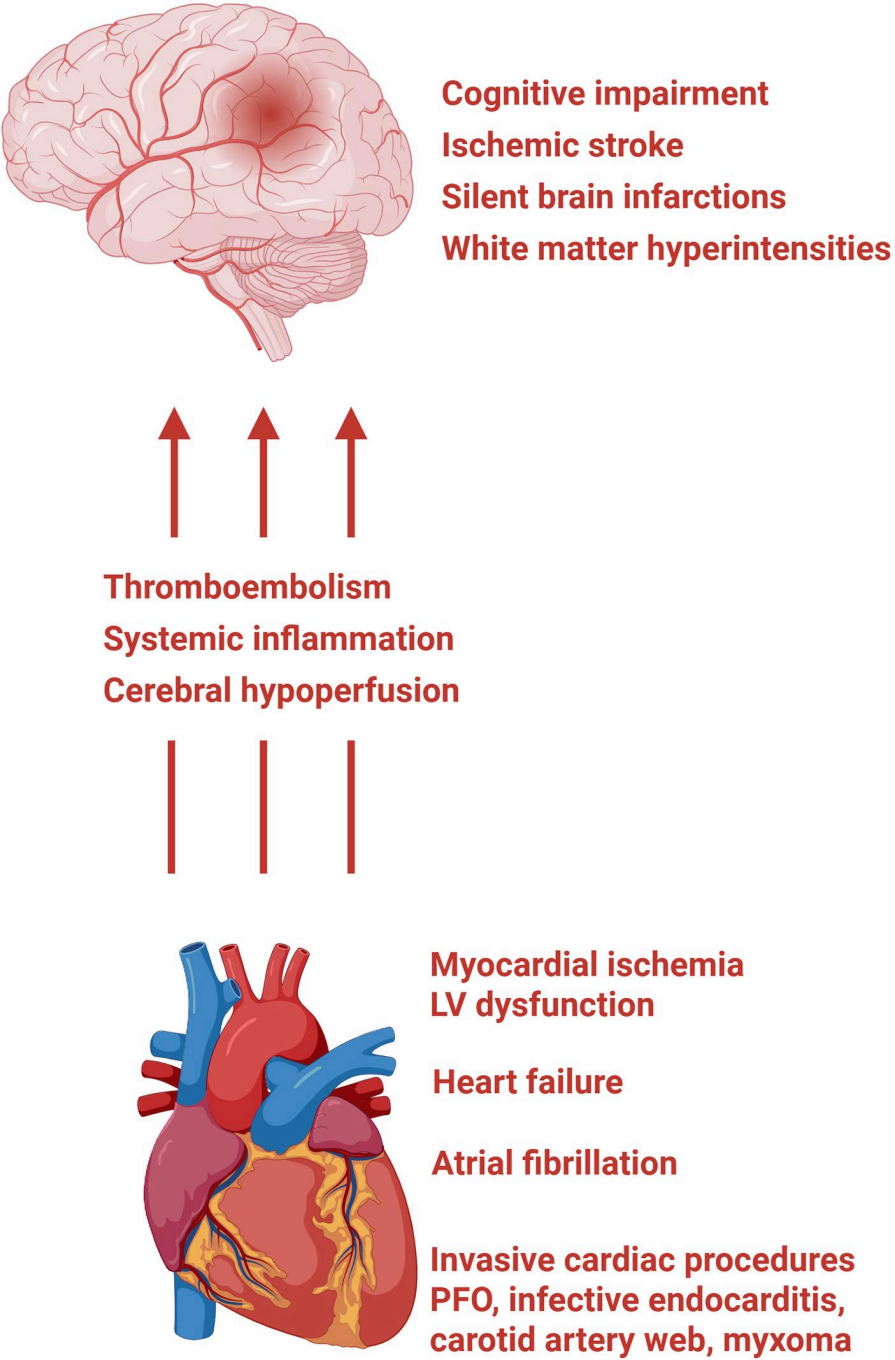
Effects of cardiac disorders on the brain. Cardiac conditions, including myocardial ischaemia and LV dysfunction, HF, AF, invasive cardiac procedures, and less common sources such as PFO, infective endocarditis, cardiac myxoma, and carotid artery web, cause cerebral injury through three converging mechanisms: thromboembolism, systemic inflammation, and cerebral hypoperfusion. Thromboembolism arises from blood stasis due to LV wall motion abnormalities and ventricular dilatation, atrial thrombus formation promoted by blood stasis within the left atrial appendage in AF, and periprocedural embolisation of thrombus, calcific debris, or atherosclerotic plaque fragments during cardiac interventions. Systemic inflammation, driven by DAMP-mediated immune activation following myocardial injury, generates a prothrombotic state, activates glial cells, and disrupts blood-brain barrier integrity, thereby amplifying neuroinflammation. Cerebral hypoperfusion results from reduced cardiac output in heart failure and from the irregular cardiac rhythm of AF, which causes repeated fluctuations in cerebral blood flow that may overwhelm autoregulatory mechanisms over time. Together, these pathways produce a spectrum of neurological consequences, ranging from clinically overt ischaemic stroke to subclinical injury detectable only on neuroimaging, including silent brain infarctions and white matter hyperintensities, as well as cognitive impairment and vascular dementia. LV, left ventricle; HF, heart failure; AF, atrial fibrillation; PFO, patent foramen ovale; DAMP, damage-associated molecular patterns.

Cardiac disease, such as MI, HF, AF, and invasive cardiac interventions, can induce brain injury through thromboembolic and inflammatory mechanisms (1, 11, 195). These processes may result in cerebral hypoperfusion, embolic events, and systemic immune activation, all of which contribute to neural damage. Secondary neuroinflammation arising from these mechanisms has been implicated in the development of ischemic stroke, cognitive impairment, depression, and vascular dementia. The bidirectional interaction between cardiac dysfunction and cerebral injury underscores the importance of considering cardiovascular disease as a major determinant of long-term neurological outcomes [Figure 10, (195–198)].

##### 2.4.3.1 Myocardial ischemia

Both acute and chronic CAD are associated with brain injury and accelerated cognitive decline (11, 14, 199, 200). A history of CAD has been associated with a 27% increased risk of dementia (201). In patients with chronic CAD, brain magnetic resonance imaging (MRI) studies have shown an estimated “brain age” approximately 2.3 years older than chronological age, suggesting accelerated cerebral aging (202). Moreover, CAD is frequently complicated by HF and AF which are strongly associated with cognitive impairment and an increased risk of dementia (13, 201).

Approximately one third of patients who experience acute MI develop cognitive impairment and behavioural disorders, including anxiety and depression (203–208). Besides impairing long-term quality of life, these neuropsychiatric conditions are associated with a significantly increased risk of MI and MACE (209, 210).

Patients with acute MI have an increased risk of stroke, particularly within the first four weeks following the acute event and over the long term. Population-based studies have reported an incidence of ischemic stroke of 2.0% within the first month and 3.8% within one year after acute MI (211–213). Stroke occurring after MI is associated with significantly higher mortality, exceeding 30% at one year, compared to 18% in those without prior MI (214, 215).

Overall, the risk of stroke is higher in patients with ST-segment elevation MI (STEMI) compared to those with non-STEMI MI. Anterior MI is particularly associated with an increased risk of aneurysm formation, regional hypokinesis, and mural thrombus development, thereby increasing the risk of embolisation. However, stroke may occur following MI involving any coronary territory and is also observed in patients with non-ischemic dilated cardiomyopathy (3).

Clinically unrecognised MI, which accounts for one-third to one-half of all MI, is also linked to a higher risk of subsequent stroke (216, 217). Unrecognised MI may result in LV scarring, regional wall motion abnormalities and LV thrombus formation. These conditions may account for a significant proportion of non-lacunar cryptogenic strokes, often classified as thromboembolic stroke of undetermined source (ESUS) (218, 219).

The incidence of LV thrombus varies widely, although it has declined in recent years due to early reperfusion therapies. Reported rates range from 4% to 39%, depending on the study population, imaging modalities, and timing of assessment (220–222). The incidence of acute ischemic stroke in patients with LV thrombus may exceed 10% (223).

The majority of ischemic stroke following MI are cardioembolic in origin and may be either clinically evident or silent, detectable only by brain MRI using diffusion-weighted imaging (DW-MRI) to identify ischemic lesions (217, 224–226). The combination of blood stasis due to LV wall motion abnormalities, endothelial ischemic injury, and hypercoagulability associated with the acute pro-inflammatory state, creates a highly thrombogenic myocardial environment favouring thrombus formation (227).

Furthermore, ischemic myocardium releases damage-associated molecular patterns (DAMPs) which activate immune cells and initiate a robust inflammatory response (228). This response is mediated by the release of cytokines, including tumour necrosis factor-alpha (TNF-α) and interleukins (e.g., IL-1, IL-16). Although this inflammatory cascade is essential for cardiac remodelling and scar formation to preserve and restore adequate myocardial function, prolonged activation may lead to a systemic inflammatory state characterized by persistent low-grade inflammation for weeks after MI (73, 229, 230).

Imaging studies have shown that myocardial inflammation can promote activation of glial cells in the brain and disrupt the integrity of the BBB, contributing to neuroinflammation and subsequent neural dysfunction (195, 231). Circulating cytokines further induce a prothrombotic state by activating endothelial cells and platelets, increasing the risk of thrombus formation within the cerebral vasculature. This may result in small brain infarcts, that contribute to cognitive impairment (232).

Furthermore, thrombi rich in activated platelets release beta-amyloid. Recurrent microthrombotic events may promote beta-amyloid aggregation within and around cerebral vessels, particularly in memory-related regions, such as the hippocampus and amygdala, leading to cerebral amyloid angiopathy. This process contributes to cognitive decline and the formation of plaques characteristic of Alzheimer's disease (233, 234).

The inflammatory response following MI also triggers coronary microvascular endothelial dysfunction, impairing vasodilation in response to increased oxygen demand and potentially leading to coronary microvascular spasm and inadequate myocardial perfusion (235). Coronary microvascular dysfunction, as part of a systemic small-vessel disease, is associated with structural alterations in the cerebral microvasculature, particularly within subcortical regions, manifesting as white matter hyperintensities on brain MRI (236–238). Clinically, cerebral small-vessel disease may present as cognitive impairment or lacunar ischemic stroke (239).

##### 2.4.3.2 Strategies to control neuroinflammation after MI

Although no therapies currently target post-MI neuroinflammation directly, several established clinical strategies may indirectly modulate neuroimmune activation by limiting myocardial injury and systemic inflammation (228). Early and effective reperfusion with primary percutaneous coronary intervention remains the cornerstone of infarct size reduction and is associated with attenuation of the inflammatory response. Smaller infarcts generate lower levels of DAMPs and pro-inflammatory cytokines, thereby reducing peripheral immune activation and subsequent neuroinflammatory signalling.

In addition, statins exert anti-inflammatory effects, suppress innate immune activation, and reduce circulating levels of C-reactive protein and interleukin-6. Antithrombotic therapies may further limit microvascular obstruction and reperfusion injury, indirectly mitigating inflammatory amplification.

Emerging evidence from anti-inflammatory cardiovascular trials, such as those evaluating colchicine (COLCOT) (240) and interleukin-1β inhibition (CANTOS) (241), suggests that systemic modulation of inflammation can improve cardiovascular outcomes and may secondarily influence neuroinflammatory pathways. However, the clinical application of these therapies is limited by an increased incidence of infections and non-CV mortality. Hence, there is the need to select high-risk patients who could benefit from a potentially harmful treatment. A further improvement will be the use of nanotechnology to produce nanoparticle drug formulations designed to selectively deliver anti-inflammatory agents to specific tissues or immune cell populations (242).

Heart failure is associated with a broad spectrum of cerebral complications, including stroke, cognitive decline, dementia, and depressive disorders (196, 243–245). Depending on the applied criteria applied, 25%–70% patients with HF exhibit cognitive impairment or dementia (201, 246, 247). The prevalence of clinically evident stroke in patients with HF ranges between 4% to 11%, with higher rates in older patients (248–250). Even in patients with preserved left ventricular ejection fraction (LVEF), subclinical LV systolic or diastolic dysfunction is associated with an increased risk of silent brain infarctions (SBI), cerebral white matter hyperintensities (WMH), and brain atrophy on MRI (251). These findings reflect underlying small-vessel disease and may represent early precursors of clinically manifest stroke (252–254).

The pathophysiological mechanisms underlying cerebral dysfunction in HF include cardiac thromboembolism and cerebral hypoperfusion secondary to reduced cardiac output. Similar to myocardial ischemia, which often precedes the development of HF, it is characterised by low-flow state promoting blood stasis, due to impaired contractility and ventricular dilatation, a prothrombotic condition driven by low-grade inflammation, and endothelial dysfunction (248, 255). Together, these conditions create a thrombogenicity cardiac environment that accounts for cardioembolic stroke in patients with HF (227, 248, 256).

Impaired LV contractile function reduces cardiac output and directly compromises cerebral perfusion, particularly when cerebral autoregulation is impaired and cerebral blood flow becomes increasingly dependent on systemic hemodynamic (257). The brain receives approximately 15% of total cardiac output and has high metabolic demand but limited energy reserves. Therefore, cerebral function is highly dependent on adequate cardiac performance. Markedly reduced LV systolic function, reflected by a decreased LVEF, is strongly associated with an increased risk of stroke (258). Conversely, chronic cerebral hypoperfusion may contribute to ANS dysfunction, further exacerbating cardiac decompensation, and initiating a self-perpetuating maladaptive neurocardiac feedback loop (259).

Prevention of cerebral injury in patients with heart failure is based on guideline-directed medical therapy (GDMT) combined with careful haemodynamic management and prevention of thromboembolic complications (245). Anticoagulation treatment is the cornerstone for prevention of cardioembolic stroke in HF with AF. Conversely, the prevention with direct oral anticoagulants (DOACs) or vitamin-k antagonists in patients with HF who remain in sinus rhythm is not well established, as potential benefits may be offset by an increased risk of major bleeding (260, 261). Neurological assessment in patients with chronic heart failure can improve early detection of cognitive impairment and silent cerebral infarction. Periodic cognitive screening and selective neuroimaging in high-risk individuals may promote timely intervention and improve strategies for secondary prevention.

Atrial fibrillation is associated with an approximately 20% lifetime risk of ischemic stroke, regardless of whether it is paroxysmal or permanent, and with a 40% increased risk of cognitive impairment, potentially related to both clinically evident and silent brain ischemia (144, 262–264). AF is also detected in up to 30% of cryptogenic strokes when prolonged cardiac rhythm monitoring is performed (265).

Traditionally, the prothrombotic risk associated with AF has been attributed to blood stasis, particularly within the left atrial appendage, which promotes thrombus formation and increases the risk of cerebral embolism. However, recent evidence suggests that the arrhythmia itself is not the sole determinant of thromboembolic risk (266). AF may arise from, or coexist with, an underlying atrial myopathy, which can contribute to stroke development independently of the arrhythmia.

Studies examining left atrial volume and strain have shown that ageing and systemic vascular risk factors, such as hypertension, pressure and volume overload, and ischemia, induce structural and functional alterations collectively termed atrial cardiomyopathy (267–269). This condition is characterised by fibrotic tissue remodelling, impaired atrial contractility, and electrophysiological disturbances. These changes result in atrial mechanical dysfunction and promote a prothrombotic state, thereby increasing the risk of cardioembolic stroke independently of manifest AF (270–273).

Other arrythmias including premature atrial contractions, paroxysmal supraventricular tachycardia, and premature ventricular contractions, have also been associated with atrial cardiomyopathy and the development of cognitive impairment and stroke (274–276). Furthermore, atrial remodelling in AF stimulates the release of DAMPs, which trigger low-grade inflammatory responses that further contribute to thrombogenesis (88, 273).

In addition to cerebral embolism as a possible mechanism underlying cognitive decline, AF may also lead to transient or chronic cerebral hypoperfusion (277). The irregular rhythm characteristic of AF leads to variability in diastolic filling times and cardiac output, resulting in repeated episodes of relative hypo- and hyper-perfusion within the cerebral microvasculature. Although cerebral autoregulation generally maintains stable cerebral blood flow across a wide range of systemic blood pressures, rapid fluctuations in cardiac stroke volume may overwhelm this compensatory mechanism. This disruption can impair normal flow-mediated vasodilation, compromise microvascular integrity, and contribute to cumulative cerebral injury over time (278).

##### 2.4.3.3 Strategies for stroke prevention

Oral anticoagulation remains the cornerstone of stroke prevention in patients with AF and is associated with significant reductions in both overt and subclinical cerebral events (144). Restoration and maintenance of sinus rhythm may further improve cerebral perfusion, reduce atrial thrombus formation, and limit microembolisation, thereby potentially attenuating subclinical brain injury. Although early randomised trials did not demonstrate a significant difference in stroke risk between rhythm-control vs. rate-control strategies, more recent evidence suggests that early rhythm-control therapy, using antiarrhythmic drugs and/or catheter ablation, may reduce the risk of stroke in patients with recently diagnosed atrial fibrillation and cardiovascular comorbidities (279). Accordingly, rhythm-control strategies may offer additional neuroprotective benefit in selected patients, whereas rate control remains an appropriate approach in clinically stable, adequately anticoagulated individuals.

##### 2.4.3.4 Invasive procedure-related thromboembolic brain ischemia

Cardiac procedures, including transcatheter and surgical valvular replacement, percutaneous and surgical coronary interventions, left atrial appendage occlusion, patent foramen ovale (PFO) closure, and AF catheter ablation, are associated with an increased risk of periprocedural cerebral ischemic injury [Table 2, (197, 280, 281)]. The extent of neurologic damage ranges from asymptomatic lesions detected only on neuroimaging to clinically disabling stroke. This risk is influenced by both procedural characteristics and patient-related comorbidities.

**Table 2:** Risk of thromboembolic stroke caused by invasive cardiac procedures.

Procedure	30-day ischemic stroke	Silent brain infarctions
Transcatheter aortic valve implantation (TAVI)	2.3–7% (290, 316–318)	∼70% (288, 289)
Surgical aortic valve replacement	5.1% (318)	58% (319)
Transcatheter mitral valve interventions	<1–2.6% (291, 320)	∼85% (321)
Mitral valve surgical interventions	3.4% (322)	n.a.
PCI	<1% (197, 323)	10% (324)
CABG	1–5% (197, 281)	n.a.
Left atrial appendage closure	0.2–1.2% (320)	52% (280)
Patent foramen ovale closure	<1% (325)	
Atrial fibrillation catheter ablation	<1 (326)	∼60% (327–329)
Left ventricular assist device (LVAD)	6–18% (197, 330)	>90%
Carotid artery revascularization	∼2% (331)	n.a.

PCI, percutaneous coronary intervention; CABG, coronary artery bypass graft.

Neurological complications are primarily caused by acute cerebral embolisation occurring during the intervention. Embolic material may consist of thrombus, calcific debris from valvular leaflets, or fragments of atherosclerotic plaque dislodged during catheter manipulation. Mechanical disruption of vascular or valvular structures may also cause endothelial injury and plaque rupture, resulting in platelet activation and thrombus formation (225, 282, 283).

An inflammatory response may also arise secondary to myocardial ischemia-reperfusion injury, which can occur following restoration of coronary flow during primary percutaneous coronary intervention or fibrinolytic therapies (284, 285). Moreover, up to three-quarters of patients undergoing transcatheter valvular procedures develop systemic inflammatory response syndrome (SIRS) which may further promote cerebral thromboembolism and device-related thrombosis (283, 286).

Although the risk of periprocedural stroke is relatively uncommon, ranging from <1% after percutaneous coronary intervention (PCI), to 18% in patients receiving a LV assist device (LVAD), SBIs are frequently detected by DW-MRI, following invasive cardiac procedures, with reported rates of up to 90% in LVAD recipients (225). The long term clinical significance of these SBIs remains uncertain, and their contribution to subsequent cognitive decline has yet to be fully elucidated (287, 288).

Strategies to reduce the risk of procedure-related cerebral injury focus on minimising embolic burden, attenuating inflammatory responses, and optimising periprocedural management. The use of cerebral embolic protection devices (CEPD) in left-sided structural heart intervention has been shown to reduce the volume of SBI but not their overall incidence (287, 289–291). Careful patient selection and comprehensive preprocedural assessment, including evaluation of atherosclerotic burden and thromboembolic risk and comorbidities, are essential to identify individuals at increased vulnerability (292).

##### 2.4.3.5 Less common sources of thromboembolic brain ischemia

Paradoxical embolism resulting from right to left shunt through PFO may account for approximately 4% of all ischemic strokes and up to 25% of embolic strokes of undetermined source, particularly in younger patients (293). The most widely accepted pathophysiological mechanism involves venous emboli bypassing pulmonary filtration by crossing the PFO into the left atrium and subsequently entering the systemic circulation. However, recent studies using high-resolution optical coherence tomography suggest that microthrombi may also arise within the PFO itself, especially in patients with a large PFO or an associated atrial septal aneurysm. These microthrombi have been described as small, irregular thrombi adherent to the endocardial surface, free-floating thrombi formed *in situ*, or localized endocardial surface irregularities (294).

Thromboembolic neurological complications may also occur in the context of infective endocarditis. Clinically overt stroke develops in approximately 25% of patients, while subclinical cerebral infarctions, detectable only by MRI, have been reported in 48%–80% of cases (295, 296). Cardiac myxoma is likewise associated with a high incidence of embolic events, with stroke reported in 26%–45% of affected individuals (297, 298).

**Figure 11:**
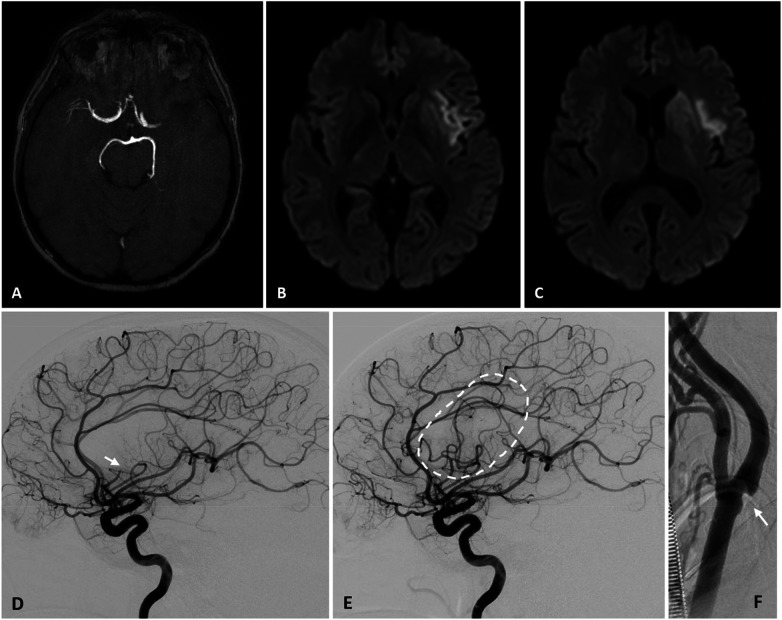
A 54-year-old woman with no cardiovascular risk factors presented with sudden-onset right-sided hemiplegia and aphasia. Emergency cerebral MR angiography revealed an occlusion of the M1 segment of the left middle cerebral artery **(A)**, resulting in a deep MCA territory infarction **(B, C)**. Intravenous thrombolysis was initiated during transfer from a peripheral hospital to the comprehensive stroke centre for thrombectomy. On arrival, initial angiographic assessment **(D)** demonstrated an occlusion of a left M2 branch (arrow). **(E)** Post-thrombectomy angiogram following a single pass with a stent retriever showed successful recanalisation and reperfusion of the downstream territory (dashed line). Final angiographic images at the left carotid bifurcation **(F)** revealed a carotid web (arrow). After excluding haemorrhagic transformation on CT at 24 h post-stroke, antiplatelet therapy with aspirin 160 mg/day was initiated for secondary prevention.

**Figure 12:**
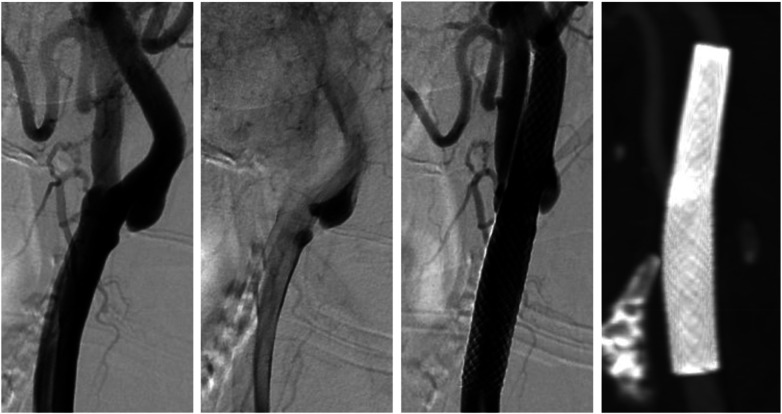
Several weeks after the ischaemic stroke, the patient underwent left carotid artery stenting. **(A)** Angiographic visualisation of the carotid web. **(B)** Contrast stagnation confirming haemodynamic disturbance caused by the web. **(C)** The stent flattens and flattens the web against the vessel wall, isolating the pouch from the bloodstream at the carotid bulb. In the following weeks, endothelialisation of the stent occurs, leading to complete exclusion and thrombosis of the pouch. **(D)** CT angiography at three-month follow-up demonstrates stent patency and complete thrombosis of the pouch.

Carotid artery web is an under-recognised cause of cryptogenic stroke in young patients, accounting for an estimated 9%–37% of such cases (299, 300). It represents a non-atherosclerotic variant of fibromuscular dysplasia, consisting of a thin, shelf-like membranous projection into the lumen of the internal carotid artery, typically at its origin (301, 302). This anatomical abnormality disrupts local hemodynamic and promotes blood stasis, facilitating thrombus formation and subsequent embolic stroke [Figures 11, 12, (303)].

## 3 Knowledge gaps and future directions

Despite substantial progress in understanding the bidirectional relationship between the heart and the brain, important knowledge gaps remain, limiting a comprehensive understanding of heart-brain interactions and their clinical implications.

Research on heart-brain axis has mainly focused on signals from the brain to the heart, while the communication from the heart to the brain has received less attention. This reverse pathway, seen in conditions such as depression and cognitive decline associated with heart failure and CAD, is not yet completely understood and requires further clinical investigation.

Another major unresolved issue concerns the limited understanding of how neurocardiac and neuroimmune interactions change across the lifespan. Age-associated alterations in autonomic regulation and immune function, together with sex-related differences, may substantially modify disease progression and treatment responsiveness, yet these factors remain insufficiently explored. Furthermore, most studies on the heart-brain axis has focused on the relationship between clinical cardiac diseases and brain disorders, while the effects of subclinical abnormalities on neurological health need to be further studied (304). Research has shown that subtle age-related changes in cardiac hemodynamic, even in the absence of CV disease, may result in impaired neurovascular coupling, brain atrophy and microvascular damage (305). In population-based settings, subclinical cardiac impairment was associated with silent lacunar infarcts and an increased risk of future stroke and dementia (306).

The autonomic imbalance in heart-brain axis dysfunction may be evidenced by reduced heart rate variability (HRV), which is strongly associated with systemic inflammation. In addition, the analysis of low-frequency oscillations in ECG recordings, termed periodic repolarization dynamics, is associated with arrhythmic events (42). However, clinical evidences are not sufficient to support their inclusion in clinical guidelines.

Identifying reliable biomarkers is essential for patient stratification and monitoring therapeutic efficacy in disorders of heart-brain axis. Currently, troponin and natriuretic peptides represent the main biomarker used in clinical practice. However, they lack specificity in distinguish between neurogenic from primary cardiac injury. Moreover, there are insufficient data to determine the proportion of stroke patients with cTn elevation that may benefit from cardiac invasive diagnostic and therapeutic measures.

New potential biomarkers whose blood levels are altered by stroke, include macrovesicles (MVs), which are a group of lipoid membrane-enclosed vesicles released by cells into the extracellular space, and microRNA, a short sequence of noncoding RNA which regulates some biological processes including responses to hypoxia, angiogenesis and inflammation, by affecting gene expression (3, 11, 58). Circulating endothelial and leukocyte-derived MVs may influence heart-brain interactions and are considered a potential biomarker for CV risk stratification (307). Circulating microRNA are being considered as diagnostic and prognostic marker in patients with stroke (308).

Therapeutic strategies targeting heart-brain pathways have not been clearly established. Modulation of the autonomic nervous system is an attractive therapeutic strategy, given the increased sympathetic activity and reduced parasympathetic tone observed in dysfunction of the heart-brain axis. Several clinical trials have investigated device-based stimulation of the vagus nerve, spinal cord, and baroreceptors, in patients with heart failure or CAD, however, with inconsistent results (309, 310). Likewise, targeting cytokines pathways which represents a central mechanism linking heart and brain pathology remain uncertain. Only low-dose colchicine has been included in the guidelines for secondary prevention when patients with chronic coronary disease or post-MI who remain at high risk despite optimal therapy, while canakinumab is not routinely recommended (311). A further improvement will be the use of nanotechnologies to produce nanoparticle drug formulation which can be delivered to specific tissue or cell populations (242).

## 4 Conclusions

The heart and the brain share complex anatomical and functional bidirectional connections. Under physiological conditions, their interplay is crucial for adapting to external stimuli and regulating internal homeostasis. In pathological conditions, damage to one of these organs causes significant pathophysiological disturbances in the other, thereby considerably worsening the prognosis.

Neuroinflammation and systemic immune activation are key mechanisms linking brain and heart disease. Stroke-related disruption of central autonomic network leads to sympathetic overactivation and persistent systemic inflammation, which contribute to myocardial injury, arrhythmogenesis, and heightened cardiovascular risk.

Conversely, cardiac disorders, such as myocardial infarction, heart failure, and atrial fibrillation can adversely impact brain structure and function These effects are mediated through shared mechanisms, including systemic inflammation, cerebral hypoperfusion, thromboembolism, and microvascular dysfunction, resulting in neurological complications such as ischemic stroke and cognitive decline.

Recognition of this bidirectional relationship may improve risk stratification, prevention strategies, and therapeutic interventions across both brain and heart disease. However, despite growing evidence, the clinical implications of heart–brain interactions remain limited in clinical practice. By synthesizing this evidence, this review aims to highlight the need for more comprehensive prognostic frameworks, integrated biomarker panels, and future trials that incorporate both cardiac and neurological outcomes.
